# DELLA-mediated gibberellin signalling regulates Nod factor signalling and rhizobial infection

**DOI:** 10.1038/ncomms12636

**Published:** 2016-09-02

**Authors:** Camille Fonouni-Farde, Sovanna Tan, Maël Baudin, Mathias Brault, Jiangqi Wen, Kirankumar S. Mysore, Andreas Niebel, Florian Frugier, Anouck Diet

**Affiliations:** 1 Institute of Plant Sciences Paris-Saclay (IPS2), CNRS, Univ Paris-Diderot, Univ Paris Sud, INRA, Univ Evry, Sorbonne Paris-Cité, Université Paris-Saclay, Bâtiment 630, Gif sur Yvette 91190, France; 2 Laboratoire des Interactions Plantes-Microorganismes (LIPM), INRA, CNRS, Castanet-Tolosan 31326, France; 3 Plant Biology Division, The Samuel Roberts Noble Foundation, Ardmore, Oklahoma 73401, United States of America

## Abstract

Legumes develop symbiotic interactions with rhizobial bacteria to form nitrogen-fixing nodules. Bacterial Nod factors (NFs) and plant regulatory pathways modulating NF signalling control rhizobial infections and nodulation efficiency. Here we show that gibberellin (GA) signalling mediated by DELLA proteins inhibits rhizobial infections and controls the NF induction of the infection marker *ENOD11* in *Medicago truncatula*. Ectopic expression of a constitutively active DELLA protein in the epidermis is sufficient to promote *ENOD11* expression in the absence of symbiotic signals. We show using heterologous systems that DELLA proteins can interact with the nodulation signalling pathway 2 (NSP2) and nuclear factor-YA1 (NF-YA1) transcription factors that are essential for the activation of NF responses. Furthermore, MtDELLA1 can bind the *ERN1* (ERF required for nodulation 1) promoter and positively transactivate its expression. Overall, we propose that GA-dependent action of DELLA proteins may directly regulate the NSP1/NSP2 and NF-YA1 activation of *ERN1* transcription to regulate rhizobial infections.

## Abstract (web summary)

Bacterial Nod Factors (NF) regulate the formation of nitrogen fixing symbiotic nodules on legume roots. Here, the authors show that gibberellins act via DELLA proteins to regulate nodulation and propose that DELLA acts with NSP2 and NF-YA1 as a transcriptional co-activator of ERN1-dependent NF signalling.

## Graphical abstract

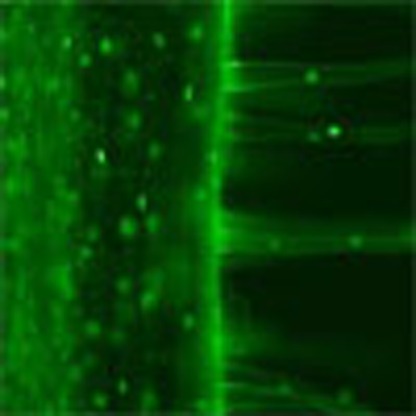

The architecture of the root system plays a crucial role in the adaptation of plant growth to environmental settings, and is consequently a key trait to maintain crop yield in response to fluctuating extrinsic conditions. Legumes, in addition to root branching through lateral roots, can develop symbiotic interactions with soil bacteria, collectively referred to as rhizobia to form another secondary root organ, the nitrogen-fixing nodule^1^. Root nodule development is initiated by a reciprocal and specific chemical dialogue between the two symbionts. Flavonoids secreted in the rhizosphere by host legume roots induce specific rhizobia to produce signalling molecules called Nod factors (NFs)^2^,^3^. The perception of NFs in the epidermis is the first step to trigger the bacterial infection of roots, eliciting root hair deformation. Tubular cell wall ingrowths containing rhizobia, called infection threads, are then formed in curled root hairs. Simultaneously, cells in root inner layers re-enter the cell cycle, giving rise to a nodule primordium. In temperate legumes such as *M. truncatula*, pericycle, endodermis and inner cortical cell divisions are induced^4^. Infection threads grow across the different root outer cell layers and subsequently ramify in the cortex. Bacteria are then released into the cells of nodule primordia, which will subsequently emerge from the root^5^,^6^. In *M. truncatula*, a persistent meristem is established at the nodule apex, allowing an indeterminate growth. Below the nodule meristem (zone I), cells elongate and are infected by rhizobia (II), and differentiate in the central nitrogen-fixing zone (III), where bacterial and plant cells co-differentiate to fix the atmospheric nitrogen and to assimilate the ammonium produced, respectively^7^.

Signalling pathways required for nitrogen-fixing symbiotic nodulation have been deciphered in the last decades^3^. Bacterial NF signals are first recognized in the root epidermis by receptor-like kinases containing LysM motives, activating a signalling cascade comprising nuclear calcium spiking. Calcium spiking is decoded by a calcium/calmodulin-dependent kinase named DMI3 (does not make infection 3) in *M. truncatula*, leading to the induction of nuclear transcriptional regulators essential to coordinate the expression of genes associated to rhizobial infection. Among these transcription factors, nodulation signalling pathway 1 (NSP1) and 2 (NSP2), nuclear factor-YA1 (NF-YA1), ethylene response factor required for nodulation 1 (ERN1) and nodule inception proteins are involved in the transcriptional activation of the infection marker *ENOD11* in the epidermis^8^,^9^,^10^,^11^,^12^,^13^,^14^,^15^,^16^. A recent model proposes that *NF-YA1* expression is NF-induced depending on DMI3 and nodule inception, and can activate *ENOD11* expression in response to NFs through the regulation of *ERN1* expression^15^, possibly within the same transcriptional complex as NSP1 and NSP2. This suggests that NSP1/NSP2 and NF-YA1 act synergistically to activate *ERN1* expression. In addition, NSP1 binds directly to the *ENOD11* promoter and this association requires NSP2 (ref. 16). Overall, this suggests that NSP1/NSP2, NF-YA and ERN1 act in combination to regulate the expression of early infection markers, such as *ENOD11* with the appropriate spatial and temporal patterns.

Beside bacterial NFs, several plant cues control nodulation progression, including phytohormones^17^. Studies based either on gain-of-function or loss-of-function mutations in a cytokinin receptor, highlight the essential role of this phytohormone in nodulation^18^,^19^,^20^,^21^. Mutations in the CRE1 (cytokinin response 1) cytokinin receptor, notably abolish the ability of rhizobia to regulate polar auxin transport locally in roots, which is correlated to the induction of nodule organogenesis^21^,^22^. In addition, this pathway directly regulates the expression of early nodulation genes such as *NSP2* that is critical for bacterial NF signalling and symbiotic nodule formation^10^,^23^. Other hormones, such as ethylene and abscisic acid negatively regulate NF signalling and nodule formation^24^,^25^,^26^. In *M. truncatula*, the ethylene-insensitive *sickle/ein2* (ethylene-insensitive 2) mutant exhibits an exaggerated number of rhizobial infection events (IEs) and a dominant-negative ABA-insensitive *abi1* (abscisic acid insensitive 1) mutant is hyperinfected, as well as hypernodulating. Gibberellins (GAs) also regulate symbiotic nodulation, even though depending on plant species, positive or negative roles were reported. Indeed, the pea GA-deficient *na* mutant showed a decreased nodulation that was restored by an exogenous GA application, suggesting a requirement of GA in nodule initiation^27^,^28^,^29^. However, in contrast to low GA concentrations (0.001 and 1 μM), exogenous treatments with a higher GA concentration (1 mM) suppressed nodulation, indicating that a positive or a negative role of GA may exist and that a tight control of GA concentration is required^29^. In addition, the *la cry* constitutively active GA signalling mutant forms fewer nodules than wild-type pea plants^28^. In *Lotus japonicus*, exogenous GA applications (1 or 0.1 μM) or overexpression of a gain-of-function *SLEEPY1*, a GA- signalling gene, resulted in an inhibition of nodulation, suppressing both epidermal infections and nodule organogenesis in the cortex^30^. Finally, in the *M. truncatula* model, no comprehensive data are available to explain GA functions in nodulation. Interestingly, a negative role of GA has recently been reported in rhizobial and arbuscular mycorrhizal symbioses, which are evolutionary related^31^, using a GA signalling loss-of-function *della1 della2* double mutant^32^.

The current model for GA signalling is that bioactive GAs are perceived by a soluble GID1 (gibberellin-insensitive dwarf-1) receptor that can interact with DELLA proteins^33^. Upon GA binding, DELLA proteins will be degraded by the proteasome through the SCF^(SLY/GID2)^ E3 ubiquitin ligase complex. The N-terminal region of DELLA proteins contains two conserved amino-acid motives, DELLA and TVHYNP, which are essential for their interaction with the GA–GID1 complex and subsequent degradation by the proteasome pathway. The C-terminal region of DELLA proteins contains a GRAS domain (named after the founding members gibberellic-acid insensitive (GAI)/repressor of GAI (RGA)/scarecrow (SCR)) that has a putative transcriptional regulatory function^33^,^34^,^35^,^36^. Depending on their ability to interact physically with different transcription factors, DELLA proteins were initially described as repressors of GA responses even though a function of the DELLA as transactivation factors was more recently proposed^33^,^37^,^38^.

In this study, we show that GAs negatively regulate *M. truncatula* nodulation and rhizobial infections depending on DELLA proteins. Indeed, the MtDELLA1 protein that is sufficient to promote *ENOD11* expression in the epidermis can interact with the critical NF signalling factors NSP2 and NF-YA1 in heterologous systems, and activate the expression of *ERN1*. This suggests a model where GA can directly regulate the NF induction of *ENOD11* expression and rhizobial infections, depending on DELLA-transcriptional complexes.

## Results

### GAs negatively regulate nodulation in *M. truncatula*

To investigate the role of GA during symbiotic nodulation in *M. truncatula*, we first assessed the effect of exogenous applications of GA_3_ (from 0.001 to 1 μM) or of the GA biosynthesis inhibitor paclobutrazol (PAC; from 0.01 to 1 μM) on nodule formation (Supplementary Fig. 1a,b). Nodule number was decreased in the presence of exogenous GA_3_ compared with the untreated condition, as a trend for lower GA concentrations (0.001 and 0.01 μM) and significantly for higher concentrations (0.1 and 1 μM; Supplementary Fig. 1a; Supplementary Table 1). Conversely, GA-inhibitory PAC treatments led to a significant increase in nodule number at 0.01 and 0.1 μM concentrations, but not at 1 μM (Supplementary Fig. 1b; Supplementary Table 1). These results therefore point towards a negative role of GA in nodulation, even though the absence of effect of the highest PAC concentration tested suggests that a fine control of GA levels is required to allow an efficient nodulation.

**Figure 1:**
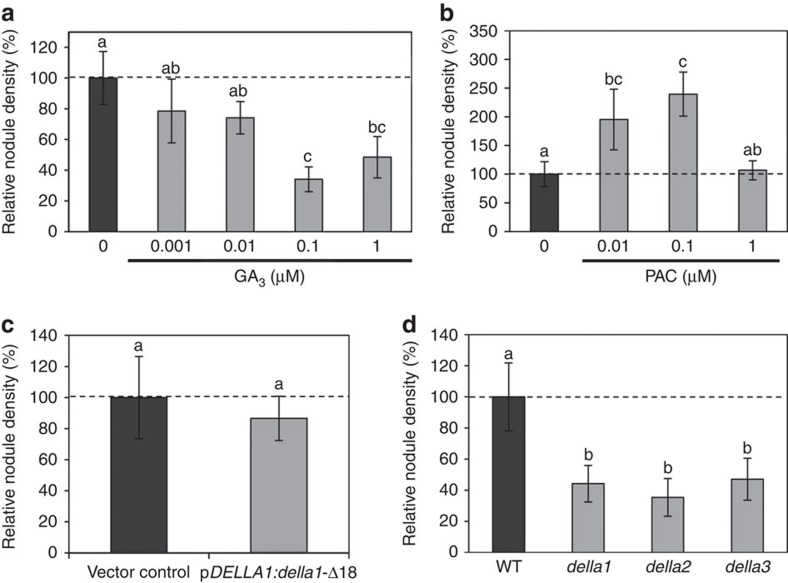
Gibberellins regulate *M. truncatula* nodulation depending on DELLA proteins. (**a**,**b**) Relative nodule density (nodule number per mg of root dry weight) of *M. truncatula* plants treated with GA_3_ (**a**) or PAC (**b**) at different concentrations. (**c**) Relative nodule density in *M. truncatula* roots expressing a dominant-active DELLA protein (p*MtDELLA1*:*della1-*Δ18) or the empty vector control. (**d**) Relative nodule density (nodule number per mg of root dry weight) of the three *della* mutants. Results are shown as percentages relatively to the untreated control (**a**,**b**,**d**) or to the empty vector control (**c**). Quantifications were performed 21 days post inoculation with *S. meliloti* (strain 1021). Dotted lines indicate a ratio of 100%. Error bars represent confidence intervals (*α*=0.05, *n*>15 plants per condition), and letters indicate significant differences based on a Kruskal and Wallis test (α<0.05) for (**a**,**b**,**d**), and a Mann–Whitney test (*α*<0.05) for **c**. One representative example out of three biological replicates is shown.

As GAs are known to regulate the plant growth^33^, we characterized root and shoot phenotypes of plants treated with the different GA_3_ or PAC concentrations described above, to exclude that the nodulation phenotypes previously observed were an indirect effect of the altered shoot and root development (Supplementary Fig. 2a–c; Supplementary Table 1). As expected, the shoots of GA-treated plants displayed increased internode lengths (Supplementary Fig. 2a). Primary root length and root dry weight of plants treated with GA_3_ concentrations ranging from 0.01 to 1 μM were slightly but significantly reduced compared with untreated plants (Supplementary Fig. 2a,b; Supplementary Table 1). Conversely, PAC treatments led to a significant increase in root length and root dry weight at 0.01 and 0.1 μM, whereas no significant change was detected at 1 μM (Supplementary Fig. 2c; Supplementary Table 1). These data suggest that GAs negatively regulate *M. truncatula* root growth and again suggest that a fine control of GA levels is required for this regulation. Similarly, shoot phenotypes were analysed and revealed that applications of GA_3_ significantly decrease shoot dry weight at high GA_3_ concentrations (0.01–1 μM) (Supplementary Table 1). In contrast, PAC treatments did not induce any significant effect on shoot dry weight (Supplementary Table 1). To evaluate the contribution of these GA-induced developmental phenotypes on the nodulation phenotype, we then determined nodule densities, corresponding to the number of nodule either per mg of root dry weight or of shoot dry weight (Fig. 1a,b; Supplementary Table 1). Both nodule densities were decreased in the presence of exogenous GA_3_ compared with the untreated condition, as a trend for lower GA concentrations (0.001 and 0.01 μM) and significantly for higher concentrations (0.1 and 1 μM; Fig. 1a; Supplementary Table 1). PAC treatments led to a significant increase in nodule densities at 0.01 and 0.1 μM (Fig. 1b; Supplementary Table 1). Altogether, these results indicate that the observed negative role of GAs in nodulation can be identified independently of shoot and root growth phenotypes.

### GA regulation of nodulation depends on DELLA proteins

**Figure 2:**
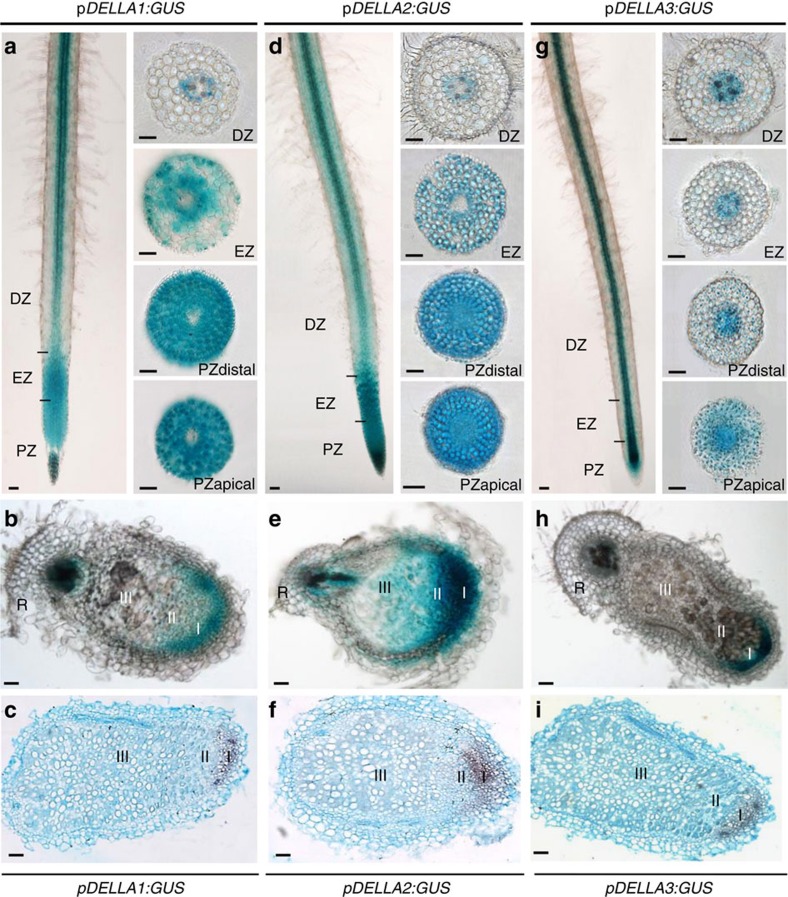
*DELLA* expression patterns in *M. truncatula* roots inoculated with rhizobium and symbiotic nodules. Histochemical analysis of GUS activity of *MtDELLA* transcriptional fusions in roots (1 day post inoculation (dpi) with *S. meliloti*) and nodules (21 dpi). Whole roots or 40 μm thick transversal root and nodule sagittal sections of *MtDELLA1:GUS* (**a**,**b**), *MtDELLA2:GUS* (**d**,**e**) and *MtDELLA3:GUS* (**g**,**h**) are shown. (**c**,**f**,**i**) are *in situ* hybridizations of *MtDELLA1* (**c**), *MtDELLA2* (**f**) and *MtDELLA3* (**i**) transcripts in 40 μm thick nodule longitudinal sections. Root zones are described as follows: DZ, differentiation zone; EZ, elongation zone; PZ, proliferation zone. Nodule zones are described as follows: I, meristem; II, infection/differentiation zone; III, nitrogen-fixation zone (according to Vasse *et al*.^7^); R, root. Scale bars, 100 μm. One representative example out of *n*=20 independent roots for histochemical analysis of GUS activity and *n*=5 nodules for *in situ* hybridization is shown.

As DELLA proteins are central repressors of GA-dependent responses^33^,^34^,^35^,^36^, we hypothesized that GAs could control the nodule formation depending on DELLAs as suggested by Floss *et al*.^32^ Three *DELLA* genes are present in the *M. truncatula* genome: *MtDELLA1* (Medtr3g065980, *M. truncatula* genome v4.0 database; http://jcvi.org/medicago/), *MtDELLA2* (contig_52215, NCBI database; http://www.ncbi.nlm.nih.gov) and *MtDELLA3* (contig_55897, NCBI database; http://www.ncbi.nlm.nih.gov). The encoded proteins MtDELLA1 and MtDELLA2 are, respectively, most closely related to the *Arabidopsis* DELLA proteins AtRGL1–3 and AtRGA/AtGAI, and are the closest relatives of the pea DELLA proteins PsLA and PsCRY^32^. The *M. truncatula* MtDELLA3 protein is more divergent compared with *Arabidopsis* and pea DELLAs, containing a non-canonical DGLLA domain^32^. The expression patterns of the three *MtDELLA* genes, based on the *M. truncatula* Gene Expression Atlas (MtGEA database, http://mtgea.noble.org/v3/; ref. 39), revealed that *MtDELLA1* and *MtDELLA2* have similar transcript levels in roots and nodules, and that *MtDELLA3* has an overall weaker expression level. In addition, the three *MtDELLA* genes do not show any strong transcriptional regulation in response to bacterial NFs^32^ (Supplementary Fig. 3a,b; ref. 40). Laser dissection of different nodule zones associated with RNAseq revealed a preferential expression of the three *DELLA* genes in the meristematic zone (I), and a lower expression of *MtDELLA1* and *MtDELLA2* in the differentiation/infection zone (II; SYMBIMICS database, https://iant.toulouse.inra.fr/symbimics/; ref. 41; Supplementary Table 2). The spatial expression pattern of the three *MtDELLA* genes was investigated using *MtDELLA* promoter:GUS (β- glucuronidase) transcriptional fusions. Under non-symbiotic and symbiotic conditions (1 day after inoculation with *Sinorhizobium meliloti*), the expression of the three *MtDELLA* genes was detected in the rhizobial infection zone (located above the root apical meristem, in the region where root hairs differentiate), in the root stele and cortex, and additionally in the epidermis for *MtDELLA1* and *MtDELLA2* (Fig. 2a,d,g; Supplementary Fig. 3c–e). The epidermal expression of *MtDELLA1* and *MtDELLA2* is consistent with transcriptomic results recently obtained by Breakspear *et al*.^40^ (Supplementary Fig. 3b). In differentiated nitrogen-fixing nodules, the *DELLA* genes were mainly expressed in the meristematic zone I (Fig. 2b,e,h), in accordance with laser dissection transcriptomic data (Supplementary Table 2). This spatial expression pattern was independently validated using *in situ* hybridization (Fig. 2c,f,i).

To determine the involvement of DELLA proteins in symbiotic nodulation, we first expressed from the *MtDELLA1* endogenous promoter a dominant version of the MtDELLA1 protein deleted of its DELLA domain (*della1*-Δ18; ref. 32), which is therefore insensitive to GA-dependent degradation. The expression of the *della1*-Δ18 gene did not reveal any significant nodulation phenotype (Fig. 1c; Supplementary Fig. 1c), even though the expression of the transgene was clearly detected (Supplementary Fig. 1f). Second, as the three *DELLA* genes are expressed in similar regions in roots and/or nodules, suggesting a potential functional redundancy (Fig. 2), we identified *Tnt1* insertional mutants affecting these three genes. *della1, della2* and *della3* mutants displayed shoot phenotypes typical of mutants having constitutively active GA responses, namely a longer stem and an early flowering phenotype^32^,^33^ (Supplementary Fig. 4). Primary root length, and root and shoot dry weights of the three *della* mutants, however, did not significantly differ from wild-type plants (Supplementary Fig. 2d; Supplementary Table 3). After *S. meliloti* inoculation, the three *della* mutants showed significantly reduced nodule numbers and densities (Fig. 1d; Supplementary Fig. 1d,e; Supplementary Table 3). Complementation assays of the three *della* mutants were performed using the respective *pMtDELLAx:DELLAx-HA* translational fusions, and both nodule number and density were restored (Supplementary Fig. 5). The identified nodulation phenotypes of single *della* mutant are therefore consistent with those obtained by Floss *et al*.^32^, with a *della1 della2* double mutant. Taken together, these results suggest that in *M. truncatula*, GAs play a negative role in nodule formation depending on a DELLA signalling pathway.

### MtDELLA1 symbiotic nuclear localization in the root epidermis

**Figure 3:**
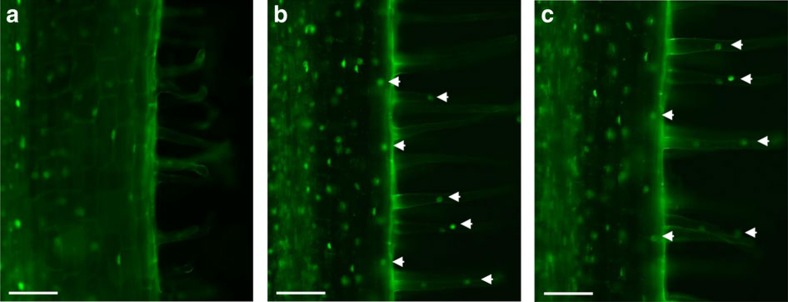
Nuclear localization of the MtDELLA1 protein under non-symbiotic and symbiotic conditions. Rhizobium infection zones of *M. truncatula* roots expressing the p*MtDELLA1*:GFP-*della1-*Δ18 construct are shown under control conditions (untreated, **a**), after a 6 h 10^−9^M Nod factors (NF) treatment (**b**) or 1 day after inoculation with *S. meliloti* (strain 1021) (**c**). White arrowheads indicate GFP signals in the nuclei of epidermal cells, including in root hairs. Scale bars, 100 μm.

To determine the subcellular localization of the three MtDELLA proteins, a transient expression assay in *Nicotiana benthamiana* was first used to ectopically express MtDELLA-GFP (green fluorescent protein) translational fusions from the 35S-CaMV promoter. The three MtDELLA-GFP fusions were detected in the nucleus, as expected from data gained in *Arabidopsis* (Supplementary Fig. 3f; ref. 42). To monitor DELLA subcellular localization in *M. truncatula* roots, we used the same translational fusions, but no GFP signal could be detected, either using the strong constitutive 35S-CaMV promoter or the endogenous *MtDELLA1* promoter, in agreement with data obtained by Floss *et al*.^32^ To overcome the fast DELLA protein turn over due to their continuous degradation by the proteasome pathway^43^, we used an N-terminal GFP translational fusion with the GA-insensitive *della1*-Δ18 variant expressed from the *MtDELLA1* promoter, previously used and functionally validated in *M. truncatula* roots^32^. This stabilized GFP-*della1*-Δ18 fusion was detected in the nuclei of cortical and stele cells (Fig. 3a). Interestingly, in response to NFs (Fig. 3b) or *S. meliloti* inoculation (Fig. 3c), the GFP-*della1*-Δ18 fusion was additionally detected in the nuclei of epidermal cells, notably in the rhizobial susceptible zone. These results indicate that at least the MtDELLA1 GA signalling factor is present in the epidermis under symbiotic conditions.

### GA and DELLAs regulate rhizobial infections in the epidermis

**Figure 4:**
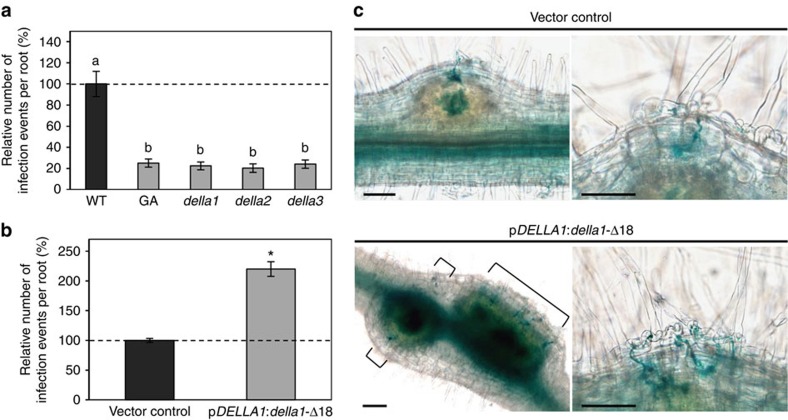
Gibberellins regulate *M. truncatula* infection depending on DELLA proteins. (**a**) Quantification of infection events per root in wild type (WT), with or without GA_3_ (1 μM) or in *della* mutants. Results are shown as percentages relatively to the WT untreated control. (**b**) Relative number of infection events in roots expressing the p*MtDELLA1*:*della1-*Δ18 construct. Results are shown as percentages relatively to the empty vector used as control. (**c**) Representative images of nodule primordia (left panels) in roots expressing the empty vector (top) or in roots expressing the p*MtDELLA1:della1*-Δ18 construct (bottom). Details of infected root hairs are shown in right panels. Square brackets indicate infection threads. Scale bars, 100 μm. In (**a**,**b**), dotted lines indicate a ratio of 100%. In all cases, quantifications were performed 7 days post inoculation with a *S. meliloti* expressing a *LACZ* reporter. Error bars represent confidence intervals (*α*=0.05, *n*>10 plants), and letters and star indicate significant differences based on a Kruskal and Wallis test (*α*<0.05) for **a** and a Mann–Whitney test (*α*<0.05) for **b**. One representative example out of two biological replicates is shown.

To determine if bacterial infection in the epidermis is affected by the GA signalling pathway, we used a *LacZ*-expressing *S. meliloti* strain that allows visualization and quantification of IEs (Fig. 4). Exogenous GA_3_ application drastically reduced IE density and accordingly, in the three *della* mutants, IE density was also strongly decreased (Fig. 4a; Supplementary Table 4). In addition, transgenic roots expressing the *della1-*Δ18 variant showed an increased IE density, even though no nodulation phenotype was previously observed (Fig. 1c; Fig. 4b,c). This result again suggests that a fine regulation of GA levels is needed to allow an efficient nodulation. Overall, these results indicate that GAs negatively regulate IEs by suppressing DELLA activity.

**Figure 5:**
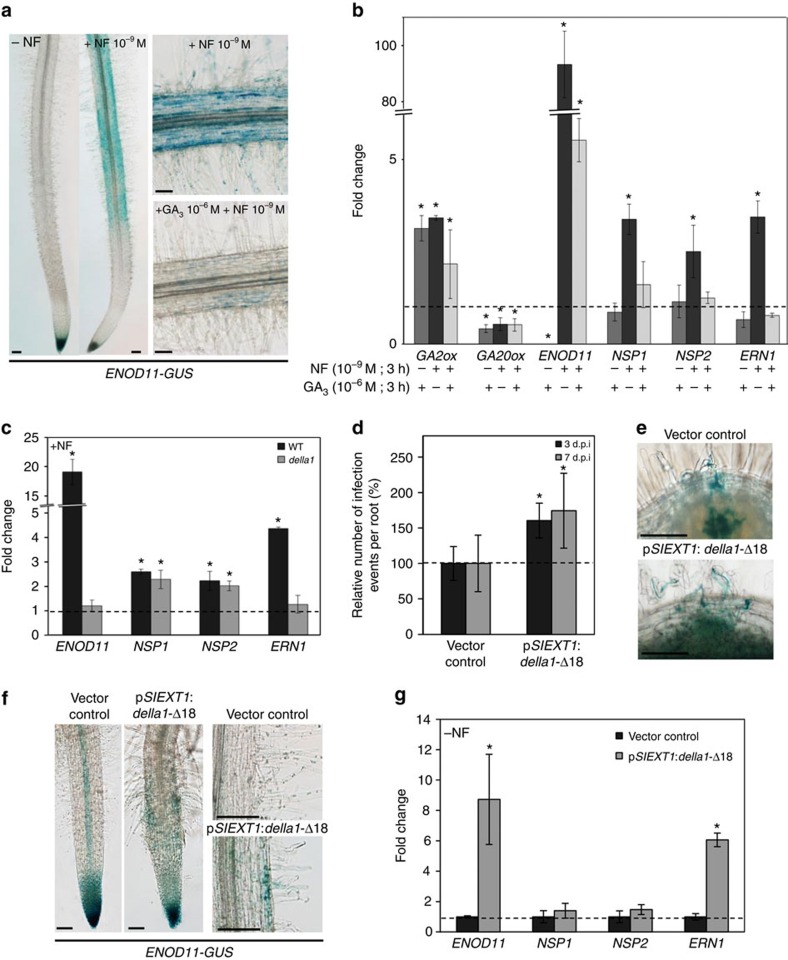
Gibberellins regulate rhizobial infection and *ENOD11* expression depending on MtDELLA1 activity in the epidermis. (**a**) Histochemical localization of GUS activity in 7-day- old transgenic seedlings expressing the rhizobial infection marker *pENOD11*:*GUS* after a NF (10^−9^M) treatment and with or without a GA_3_ (1 μM) pre-treatment. (**b**) Expression of *GA2ox, GA20ox, NSP1, NSP2*, *ERN1* and *ENOD11* in WT roots after a NF (10^−9^M) treatment, and with or without a GA_3_ (1 μM) pre-treatment. (**c**) Expression of the *ENOD11* infection marker and of *NSP1, NSP2* and *ERN1* after a NF (10^−8^M) treatment in the WT and in *della1* mutant roots. (**d**) Relative number of infection events in p*SlEXT1*:*della1-*Δ18 roots, at 3 and 7 days post inoculation with *S. meliloti* (strain 2011) expressing the *LACZ* reporter. Results are shown as percentages relatively to the empty vector control. (**e**) Representative images of infection threads visualized by staining of *S. meliloti* (strain 2011) expressing the *LACZ* reporter in roots transformed with the vector control (top) or the p*SlEXT1*:*della1-*Δ18 (bottom) construct. (**f**) Histochemical localization of GUS activity in transgenic roots expressing p*ENOD11*:*GUS*, transformed with the empty vector control or the p*SlEXT1*:*della1*-Δ18 construct. Details of root hairs are shown in right panels. (**g**) Expression of *ENOD11, NSP1, NSP2* and *ERN1* in non-inoculated roots expressing p*SlEXT1*:*della1*-Δ18 or the vector control. In **b**,**c** and **g**, transcript levels are normalized relatively to untreated control roots to show fold changes and the dotted line indicates a ratio of 1. In **d**, results are shown as percentages relatively to the empty vector control and dotted line indicates a ratio of 100%. Error bars represent s.d. in **b**,**c** and **g**, and confidence intervals (*α*=0.05, *n*>15 plants per condition) for **d**. In all cases, the asterisks indicate significant difference compared with the control based on a Mann–Whitney test (*α*<0.05). One representative example out of two biological replicates is shown. Scale bars, 100 μm (**a**,**e**).

To pinpoint molecular mechanisms that could explain this phenotype, we analysed the effect of GAs on the expression of one of the earliest NF-induced infection marker: *ENOD11* (ref. 8). Transgenic plants expressing a p*ENOD11:GUS* transcriptional fusion were treated with NF for 3 h, with or without a 3 h GA_3_ pre-treatment. As previously described^8^, the NF-induced *ENOD11* expression was detected in epidermal cells of the rhizobial infection zone (Fig. 5a). Interestingly, a pre-treatment with GA_3_ inhibited the NF induction of *ENOD11* (Fig. 5a). To independently and quantitatively confirm this result, the expression of *ENOD11* was determined by real-time PCR with reverse transcription (RT–PCR) in roots challenged with the same treatments. The efficiency of the GA_3_ treatment was determined by monitoring the expression of a GA catabolic (*GA2ox*) and of a GA biosynthetic (*GA20ox*) gene, which are, respectively, up- and downregulated by the exogenous GA_3_ treatment, as a result of a negative feedback (ref. 44; Fig. 5b). As previously observed, the NF-induced expression of *ENOD11* was drastically reduced in GA_3_ pre-treated roots (Fig. 5b). Because MtDELLA1 was detected in the nuclei of epidermal cells in the rhizobial susceptible zone under symbiotic conditions (Fig. 3b,c), and because the expression of the *della1-*Δ18 variant increased IE density (Fig. 4b), we tested if the NF induction of *ENOD11* depended on MtDELLA1. The NF regulation of *ENOD11* was strongly reduced in the *della1* mutant (Fig. 5c). Together, these results indicate that in agreement with the previously observed infection and nodulation phenotypes, the early NF-dependent *ENOD11* expression is rapidly regulated by GAs depending at least on MtDELLA1.

To further test whether MtDELLA1 has a role in symbiosis in the epidermis, the *della1*-Δ18 dominant-active protein was expressed from a p*SlEXT1* promoter (*Solanum lycopersicum* extensin1), previously demonstrated to be specifically active in the epidermis of *M. truncatula* roots^45^. Either 3 or 7 days after inoculation with *S. meliloti*, IE density was significantly increased in these roots (Fig. 5d,e; Supplementary Table 4), indicating that the ectopic expression of a dominant version of MtDELLA1 in the epidermis is sufficient to promote rhizobial infection. Accordingly, the ectopic expression of *della1*-Δ18 in the epidermis of stable transgenic plants expressing the p*ENOD11:GUS* transcriptional fusion revealed an activation of the *ENOD11* promoter in epidermal cells of the rhizobial infection zone in the absence of rhizobia or NFs (Fig. 5f). This result was independently and quantitatively confirmed by real-time RT–PCR (Fig. 5g), indicating that the dominant active form of a DELLA protein expressed in the epidermis is sufficient to spontaneously activate a symbiotic infection pathway related to *ENOD11*. This suggests that the GA regulation of symbiotic infections and NF signalling depends on the cell autonomous action in the root epidermis of at least MtDELLA1.

### MtDELLA1 interacts with NSP2 and NF-YA1 and activate *ERN1*

As GAs inhibit the NF induction of the *ENOD11* symbiotic infection marker, the expression of which directly depends on the previously identified NF signalling genes *NSP1*, *NSP2* and *ERN1* (refs 10, 11, 12, 13, 14), we then investigated if GAs could affect their expression. The NF induction of *NSP1, NSP2* and *ERN1* was inhibited by a GA_3_ pre-treatment (Fig. 5b), suggesting that GAs negatively regulate nodulation potentially by repressing these key NF signalling genes. We therefore tested if MtDELLA1 was involved in this regulation. The NF induction of *NSP1* and *NSP2* expression was maintained in the *della1* mutant, whereas the NF induction of *ERN1* and *ENOD11* was strongly reduced (Fig. 5c). This suggests that the MtDELLA1-dependent induction of *ENOD11* expression by NFs may require activation of ERN1. Interestingly, in the absence of rhizobia or NFs, the ectopic expression of *della1*-Δ18 in the epidermis previously shown to induce *ENOD11* expression, was sufficient to activate *ERN1* as well, but not *NSP1* and *NSP2* (Fig. 5g). These results indicate that at least MtDELLA1 is required in the epidermis to activate both *ERN1* and *ENOD11* expression. Accordingly, a chromatin immunoprecipitation (ChIP) experiment performed in *M. truncatula* roots 1 day post inoculation with rhizobium indicated that pMtDELLA1:MtDELLA1 can associate with the *ERN1* promoter (Supplementary Fig. 6).

**Figure 6:**
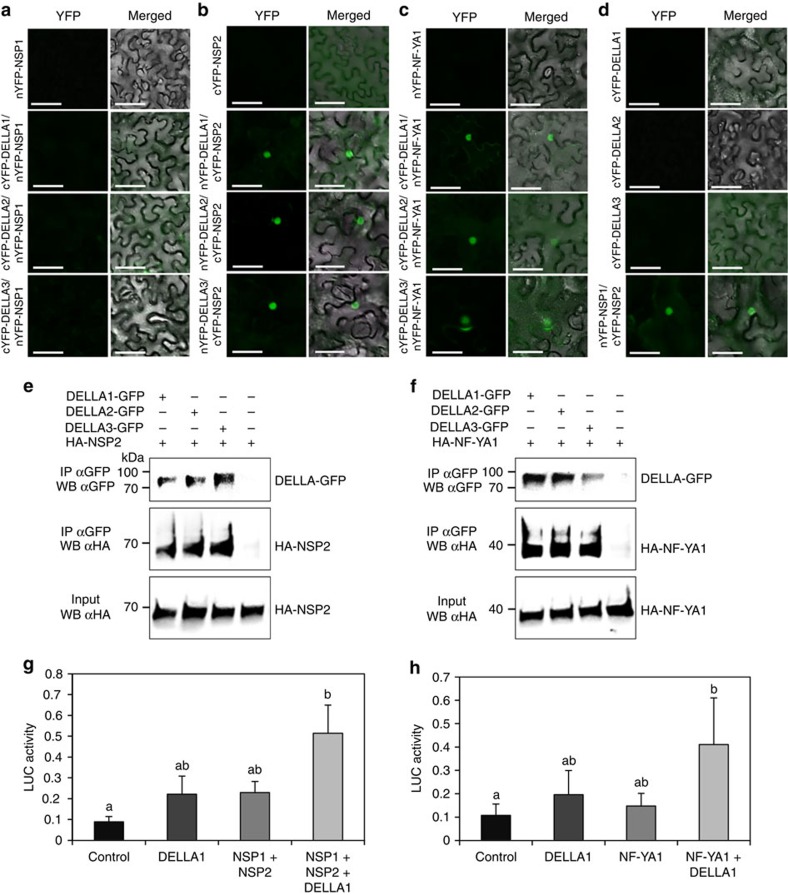
DELLAs can interact with the NF signalling transcription factors NSP2 and NF-YA1, and MtDELLA1 can enhance *ERN1* transcriptional activation. (**a**–**d**). Bimolecular fluorescence complementation analysis of the interaction between nYFP-NSP1 and cYFP-MtDELLA1 to 3 (**a**), cYFP-NSP2 and nYFP-MtDELLA1 to 3 (**b**), and nYFP-NF-YA1 and cYFP-MtDELLA1 to 3 (**c**) in *N. benthamiana* leaves. The negative controls (each construct alone) are shown in the different panels, as well as the nYFP-NSP1-cYFP-NSP2-positive control (**d**). In all panels, YFP fluorescence alone (left) and bright-field merged images (right) are shown. Scale bars, 100 μm. (**e**,**f**) Co- immunoprecipitation of MtDELLA1-GFP, MtDELLA2-GFP or MtDELLA3-GFP proteins expressed in *N. benthamiana* leaves with either the HA-NSP2 protein (**e**) or the HA-NF-YA1 protein (**f**). Blots were revealed with an anti-GFP (upper panel) or an anti-HA (middle and lower panels) antibody. Inputs were used as loading controls. (**g**,**h**) Transactivation assays in *A. thaliana* protoplasts of the *pERN1:LUC* construct by NSP1/NSP2 (**a**) or NF-YA1 (**b**) with or without MtDELLA1. An empty vector is used as negative control. Results represent the means of two biological replicates, error bars represent the s.e., and letters indicate significant differences (*α*<0.01) using a Kruskal and Wallis test.

The NSP1/NSP2 GRAS and the NF-YA1 transcription factors have been previously shown to directly bind the *ERN1* promoter to positively regulate its expression^13^,^14^,^15^,^16^. As we now demonstrated that at least MtDELLA1 is required to induce *ERN1* and *ENOD11* expression in response to NFs (Fig. 5c), and as DELLA proteins have been previously shown to interact with transcription factors^33^, we then tested if DELLA proteins were able to interact with the NSP1, NSP2 or NF-YA1 transcription factors (Fig. 6). Using bimolecular fluorescence complementation (BiFC), interactions in the nuclei could be detected between the three DELLA proteins and NSP2 (Fig. 6b), but not NSP1 (Fig. 6a). As previously described^16^, the NSP1 BiFC construct revealed an interaction with NSP2 (Fig. 6d), indicating its functionality and thus revealing specificities among GRAS protein interactions. In addition, a nuclear interaction between the three DELLA proteins and the NF-YA1 transcription factor was detected (Fig. 6c). Interactions between the three DELLA proteins, and the transcription factors NSP2 and NF-YA1 were further independently confirmed by co-immunoprecipitation (Co-IP) assays in *N. benthamiana* leaves (Fig. 6e,f). As NF-YA1 and NSP2 proteins may be recovered simultaneously in a MtDELLA IP, this suggests that these protein interactions might not be exclusive (Supplementary Fig. 7). Overall, these results indicate that *M. truncatula* DELLA proteins can interact, at least in heterologous systems, with two critical NF signalling transcription factors, NF-YA1 and NSP2, known to positively regulate the *ERN1* symbiotic expression.

We then finally investigated whether MtDELLA proteins could influence the transcriptional activation of *ERN1* mediated by the NSP1/NSP2 or NF-YA1 transcription factors (Fig. 6g,h). Interestingly, transient activation assays in *Arabidopsis* protoplasts revealed that the expression of a p*ERN1:LUC* reporter construct is significantly increased when *NSP1/NSP2* (Fig. 6g) or *NF-YA1* (Fig. 6h) are co-transfected with *MtDELLA1*. These results suggest that at least MtDELLA1 is able to function as a transcriptional activator of *ERN1* expression in protoplasts, which can potentiate the action of the NSP1/NSP2 and NF-YA1 transcription factors. Overall, based on these results, we propose that MtDELLA1 protein positively regulates the expression of *ERN1*, depending on its ability to interact with key NF signalling transcription factors binding the *ERN1* promoter, to subsequently regulate the *ENOD11* expression and the progression of rhizobial infections.

## Discussion

In legumes, root symbiotic nodulation is tightly and dynamically regulated to ensure that the extent of rhizobial infections, nodule number and ultimately nitrogen-fixation activity are coordinated with the availability of other soil nitrogen sources and with the carbon provided from shoots. This control is notably exerted by several plant hormones that affect different stages of symbiotic nodulation. Most plant hormones previously reported to regulate rhizobial infection and/or NF signalling in the epidermis have been described to act at or upstream of calcium spiking. For instance, ethylene, abscisic acid and jasmonic acid inhibit the rhizobial infection by negatively regulating the multiple epidermal responses^24^,^25^,^26^. Whereas ethylene and abscisic acid act in parallel pathways to regulate early infection stages, jasmonic acid and ethylene act synergistically to inhibit the nodulation, but these two hormones have an antagonistic role in the regulation of the calcium spiking^24^,^25^,^26^,^46^. This indicates that complex crosstalk between several hormones control NF signalling upstream of calcium spiking, even though molecular mechanisms involved remain unknown. Consistent with the Maekawa *et al*.^30^ study in *L. japonicus*, we show in this study that the GA phytohormones play a negative role in *M. truncatula* nodulation at an early step of the NF signal transduction pathway, downstream of calcium spiking. This regulation may involve an interaction between the GRAS transcriptional regulators DELLA, acting in GA signalling, and the NF signalling transcription factors NSP2 and NF-YA1, highlighting potential crosstalk between these two signalling pathways.

Previous studies already revealed that GAs play an important role in legume nodulation, either positive or negative depending on legume species and experimental approaches used^17^. On the one hand, in *Sesbania rostrata* lateral root-based nodulation, a GA biosynthesis inhibitory treatment with chlormequat chloride decreased nodulation; in pea, exogenous GA_3_ applications at low concentrations (0.001 and 1 μM) increased nodulation and a 1 μM GA_3_ treatment was able to rescue the reduced nodulation phenotype of the dwarf GA biosynthetic *na* mutant^27^,^47^. In addition, GA_3_ applications in *L. japonicus* induced the formation of pseudo-nodule structures in the absence of rhizobia^48^. These data suggest that GAs act positively in nodulation and notably in organogenesis. On the other hand, the application of exogenous GA_3_ (10 μM) inhibited root hair curling nodulation in *S. rostrata* and infection in *L. japonicus,* and a high GA_3_ concentration (1 mM) decreased pea nodulation^27^,^30^,^47^. In *L. japonicus*, exogenous GA_3_ applications ranging from 0.001 to 1 μM inhibited root nodule formation and conversely, treatments with a GA biosynthesis inhibitor increased nodule number^30^. Accordingly, a gain-of-function mutation of the *L. japonicus* GA signalling F-box protein SLEEPY1 (SLY1)^30^ and a pea DELLA loss-of-function *la cry* double mutant^28^ show a reduced number of nodules, indicating that the DELLA-dependent GA signalling pathway positively regulates nodulation. This suggests a negative role of GAs in nodulation, notably in rhizobial infection. Accordingly in *M. truncatula,* exogenous applications of GA_3_ (in the same concentration range as tested in *L. japonicus*) and mutation in *della* genes decreased the number of nodules and epidermal infections^32^ (and this study), whereas low PAC concentrations increased the number of nodules and the expression of the *della1*-Δ18 variant from the *MtDELLA1* promoter increased the number of infections. This therefore reveals a negative role of GA and a positive role of DELLA proteins in *M. truncatula* nodulation and rhizobial infection. Higher PAC concentrations (1 μM) or *della1*-Δ18 roots however did not lead to the formation of an increased number of nodules in *M. truncatula*, as it would be expected. Altogether, these data suggest that a fine-tuned GA ‘window' is required for an efficient nodulation, which may depend on species and approaches used^17^,^27^,^28^,^29^.

Several transcriptomic analyses revealed that the expression of GA metabolic genes, such as *GA2ox* catabolic and *GA20ox* biosynthetic genes, are regulated during early nodulation a few hours after rhizobium or NF application^40^,^47^,^49^,^50^ (and this study), as well as in the evolutionary-related mycorrhizal endosymbiosis^51^,^52^,^53^, where a significant increase of endogenous GA levels is observed during colonization^54^. In *L. japonicus*, depending on GA concentrations, positive or negative effects on the expression of mycorrhizal infection genes have been observed^55^,^56^, and in pea, a GA_3_ treatment decreased hyphal colonization and arbuscule formation, but increased hyphal branching in cortical cell layers^52^,^53^. These results indicate a dual function of GA in the regulation of endomycorrhiza symbioses, which is reminiscent of results obtained in symbiotic nodulation. Several recent studies also highlighted that a shared GA/DELLA regulation may exist between NF and Myc factor signalling pathways. Indeed, pea *la cry* and *M. truncatula della1 della2* double mutants have both a drastic decrease in arbuscule formation, associated to a reduction of hyphal growth in pea, but not in *M. truncatula*^32^,^57^. Similarly in rice, a mutation of the OsSLR1 DELLA protein induced a severe reduction of hyphaes, arbuscules and vesicles, whereas hyphopodia formation seemed unaffected^58^. All these studies then point to a positive role of DELLA proteins in the regulation of arbuscule differentiation and/or AM colonization, as observed for nodulation and rhizobial infection.

Accordingly to the transcriptomic data, *in situ* and GUS transcriptional fusions revealed that the three *MtDELLA* genes have similar expression patterns in the root rhizobial infection zone and in nodules, suggesting a potential redundancy, similarly as in *Arabidopsis* where the five AtDELLA proteins have partially overlapping functions^33^. Nodulation phenotypes of *M. truncatula* mutants affecting each of the three *MtDELLA* genes have a similar strength, although MtDELLA3 has a modified DELLA domain (DGLLA) and a weaker expression level than *MtDELLA1* and *MtDELLA2*. Noteworthy, GA-related shoot phenotypes observed in each single *della* mutant also have a similar strength, even though *MtDELLA3* expression is weaker than *MtDELLA1* and *MtDELLA2* also in shoots. This may indicate that the absence of one DELLA may affect the function of the others. The nodulation phenotype of the three single *della* mutants reported in this study appears to be stronger than the one previously observed in a *della1 della2* double mutant^32^. These discrepancies between studies may be explained by the use of different *della1* mutant alleles, diverse rhizobial strains, inoculation and plant growth conditions, as well as ways to score nodulation phenotypes. Accordingly, strong GA-related shoot phenotypes observed in our study in single *della* mutants were not reported elsewhere^32^. Nevertheless, both studies identified a low nodulation phenotype, independently of *della* mutant alleles and combinations used^32^ (and this study), further indicating a positive role of DELLA in *M. truncatula* early nodulation.

Interestingly, the GFP-*della1*-Δ18 translational fusion revealed a nuclear accumulation of the GFP signal in epidermal cells under symbiotic conditions, although no GFP signal was detected in the epidermis under non-symbiotic conditions. Whereas we do not exclude that a DELLA protein level below the GFP detection limit exists, an alternative interpretation would be that DELLA proteins might be recruited in the epidermis in response to rhizobium and NFs, implying that such regulation would be independent of the NSP/ERN NF signalling pathway. Strikingly, transcriptional analyses in *M. truncatula* also revealed that most of the NF signalling genes, such as *NSP1, NSP2, ERN1* and *NF-YA1* are themselves rapidly induced by NFs^10^,^12^,^13^,^14^,^15^, including in root hairs^40^. DELLAs would therefore be another example of a critical NF signalling component, which regulation depends on its own pathway, even though in the case of DELLA proteins, this regulation may involve post-transcriptional regulations. Interestingly, DELLA function in arbuscular mycorrhizal symbiosis was proposed to act non-cell autonomously from the vascular tissues and endodermis to enable arbuscule formation in a distant cortex layer, and a movement of DELLA proteins was hypothesized to explain this non-cell autonomous effect^32^.

During rhizobial infection, a specific transcriptional cascade in the epidermis leads to the activation of the infection marker *ENOD11* (ref. 9). In *L. japonicus*, Maekawa *et al*.^30^ demonstrated that GAs negatively regulate the NF-induced expression of *NSP1* and *NSP2.* Our study shows that the NF induction of the *ERN1* expression, acting upstream of *ENOD11*, is strongly inhibited by a GA treatment. In addition, the NF-induced *ERN1* expression was lost in the *della1* mutant and the ectopic expression of the stabilized dominant-active *della1-*Δ*18* variant in the epidermis was able, in the absence of bacterial or NF signals, to induce *ERN1* expression. This indicates that the epidermal expression of MtDELLA1 under non-symbiotic conditions is sufficient to activate at the molecular level an infection-related pathway. As it was recently proposed that NSP1/NSP2 and NF-YA1 transcriptional complexes directly participate in the NF regulation of *ERN1* to subsequently activate *ENOD11* expression^14^,^15^, these results suggest that the induction of *ERN1* expression requires MtDELLA1-dependent activation of NF-YA1 and/or NSP1/2 complexes.

DELLA proteins can recruit different types of transcription factors to regulate gene expression with a high spatiotemporal specificity^33^. We show in this study that MtDELLA1, MtDELLA2 and MtDELLA3 can interact with NSP2 and NF-YA1. It should be however noted that these protein interactions were only detected in a heterologous system, and not in *M. truncatula* roots. We could nevertheless demonstrate in *M. truncatula* roots that MtDELLA1 expressed from its own promoter can bind the *ERN1* promoter, and can promote its expression in an heterologous transactivation assay. Altogether, based on these results, we propose that, in addition to NSP2 and NF-YA1 transcription factors, DELLA proteins act as positive transactivation factors of *ERN1* expression. Related functions were recently described in *Arabidopsis* and rice^33^,^37^,^38^. Indeed, while DELLA proteins were mainly considered until recently as having a repressive role, the AtRGA protein was shown to form a complex with an IDD protein (indeterminate domain) to upregulate the expression of *SCL3* (scarecrow-like 3)^37^. In addition, GRAS transcription factors related to NSP2 have also recently been identified as interactors of a DELLA protein in rice^58^. As mentioned previously, GA and DELLA proteins seem to have similar functions in early rhizobial and endomycorrhizal interactions. At the molecular level, the unique rice DELLA, OsSLR1 (slender rice 1) interacts with the GRAS protein DIP1 (standing for DELLA interacting protein 1), which is positively involved in the endomycorrhiza symbiotic interaction. DIP1 interacts with another GRAS transcription factor, RAM1 (reduced arbuscular mycorrhization 1) required for the activation of the *RAM2* mycorrhizal-induced gene, and thus for symbiotic fungi colonization^58^,^59^.

A RAM1/DIP1/SLR1 GRAS protein complex would then be required for the positive regulation of mycorrhizal-associated gene expression and fungi colonization. In the nodulation context, it was previously documented that NSP1 interacts with NSP2 and that this interaction is required for nodulation^16^. A NSP1/NSP2/DELLA GRAS protein complex may then positively regulate gene expression controlling rhizobial infection and nodulation. A parallel has recently been established at the molecular level between the regulation of *ERN1* and *RAM2* expression in response, respectively, to nodulation and mycorrhizal signals^3^, which suggests that NSP1 may be functionally equivalent to RAM1. Interestingly, DELLA proteins interact with DIP1 but not RAM1 (ref. 58), consistent with our results, in which we did not find interaction between DELLA proteins and NSP1, suggesting specificities in GRAS protein interactions. It remains to be tested if the DELLA/NSP2 interactions identified here are relevant for the mycorrhizal symbiosis. In this latter case, another GRAS protein different from NSP2 was identified as a DELLA interactor, suggesting that other GRAS proteins may also interact with DELLAs in the nodulation context. Our study additionally reveals that other types of NF-related transcription factors may participate in the same transcriptional complex, such as NF-YA1. Capturing the kinetics and spatial locations of these different protein interactions will be essential to determine when and where these various transcriptional complexes can be formed and on which target they act. In addition, overlaps between DELLA-transcriptional complexes recruited for rhizobial and endomyccorhizal early signalling pathways, and their relevance for the regulation of NF versus mycorrhizal signalling and the progression of symbiotic infections also remains to be determined.

## Methods

### Materials and treatments

Seeds of the *M. truncatula* genotypes Jemalong A17 or R108 were used in this study. The *della1* (NF12399), *della2* (NF4302) and *della3* (NF10539) mutant lines were identified by a PCR-based reverse screening^60^ of *M. truncatula Tnt1* insertion population^61^, generated in the R108 genotype, at the Noble Foundation (http://bioinfo4.noble.org/mutant/database.php). Mutant plants were genotyped by PCR using specific primers to amplify the endogenous loci, which were also combined with the transposon-specific primer LTR4 or LTR6 (Supplementary Table 5 for primer combinations and sequences) and backcrossed twice. The *pENOD11*:*GUS* line was initially identified in Journet *et al*.^8^. Seeds were scarified and sterilized as described in Gonzalez-Rizzo *et al*.^18^.

For *in vitro* nodulation experiments, germinated seeds were grown vertically on a Fahraeus medium without nitrogen^62^ with 1.5% Bacto-Agar (Gibco) for 7 days in chambers at 24 °C under long-day conditions (16 h light at 150 μE light intensity/8 h dark), and then inoculated with a *S. meliloti* strain 1021 suspension (OD600nm=0.07) for 1 h. A derivative *S*. *meliloti* strain 2011 (ref. 63) carrying the pXLGD4 plasmid containing a Pro_Δ*ala*_:*LACZ* transcriptional fusion was additionally used for LacZ staining experiments.

For *in vitro* pharmacological treatments, germinated seeds were grown as described above on a growth paper (Mega International, http://www.megainternational.com/index.htm). After 2 days, the growth paper with the plants was transferred on a fresh Fahraeus medium without nitrogen, supplemented or not with increasing concentrations of GA_3_ (0.001–1 μM, Sigma-Aldrich) or PAC (0.01–1 μM, Sigma-Aldrich).

To generate material for real-time quantitative RT–PCR (RT–qPCR) expression analyses, twenty germinated seedlings were placed on a grid in a magenta box with a low-nitrogen liquid medium (‘i'; Blondon, 1964) and grown in a shaking incubator (125 r.p.m.) at 24 °C under long-day conditions (16 h light, 150 μE/8 h dark). After 4 days, seedlings were treated or not with 1 μM GA_3_ (Sigma-Aldrich) and maintained under the same growth conditions for various incubation times (0 and 3 h). Similarly, NF purified from *S. meliloti* were used at 10^−9^M for 3 h, after or not, an incubation for 3 h with 1 μM GA_3_. In parallel, mock experiments were performed, by collecting untreated roots across the kinetic (0 and 3 h). Roots collected at the indicated time points were immediately frozen in liquid nitrogen.

### Gene expression analysis

Transcriptomic data were retrieved from the *M. truncatula* Gene Expression Atlas (MtGEA database, http://mtgea.noble.org/v3/; ref. 39), or from the SYMBIMICS website (https://iant.toulouse.inra.fr/symbimics/; ref. 41).

For RT–qPCR experiments, total RNA was extracted from frozen roots using the RNeasy plant mini kit (Qiagen). First-strand complementary DNA (cDNA) was synthesized from 1 μg of total RNA using the Superscript II First- Strand Synthesis System (Invitrogen). Primer design was performed using Primer3 software (http://frodo.wi.mit.edu/cgi-bin/primer3/). Primer combinations, showing a minimum amplification efficiency of 80%, were used in RT–qPCR experiments (Supplementary Table 5). Specificity was checked both using a melting curve and by sequencing the PCR amplicons. RT–qPCR reactions were performed using the LightCycler480 SYBR Green I Master Kit on a LightCycler480 apparatus according to manufacturer's instructions (Roche). Reference genes (*RBP1* and *ACTIN11*) were selected using the geNorm software^64^.

For *in situ* hybridizations, the protocol described in Bustos-Sanmamed *et al*.^65^ was used on an Intavis InsituPro automat (http://www.intavis.com/en/), on nodules 21 days after *S*. *meliloti* (strain 1021) inoculation. Antisense RNA probes corresponding to *MtDELLA1*, *MtDELLA2* and *MtDELLA3* were generated. Sense and antisense RNA probes corresponding to a carbonic anhydrase (Mt*CA1*) gene were included as negative and positive controls, respectively^21^. All primers used are shown in Supplementary Table 5.

### Cloning procedures

For transcriptional GUS fusions, available sequences upstream of *MtDELLA1*, *MtDELLA2* or *MtDELLA3* start codon (1,987, 1,121 or 2,431 bp, respectively) were amplified by PCR with a Pfx polymerase (Invitrogen) using primers listed in Supplementary Table 5. PCR fragments were first cloned into the pTOPO-ENTRY/D vector (Invitrogen) and transferred using the Gateway technology (LR recombination) into the pkGWFS7 vector (http://www.psb.ugent.be/gateway/index.php) carrying a *GUS* sequence downstream of the cloning site. For *della* mutants complementation assays, the p*MtDELLA*x:DELLAx-HA constructs were obtained using the MultiSite Gateway Cloning System (Invitrogen) and the destination vector pK7m34GW^66^. For translational fusions, *MtDELLA1*, *MtDELLA2* or *MtDELLA3* cDNAs were amplified by PCR (primer sequences shown in Supplementary Table 1), first cloned into the pTOPO-ENTRY/D vector (Invitrogen), and subsequently in the pK7wGF2 vector containing a p35 S-GFP cassette (http://www.psb.ugent.be/gateway/index.php). For 35S:HA-NF-YA1, we used the clone generated in Laloum *et al*.^15^, and for 35S:HA-NSP1 and 35S:HA-NSP2 in Cerri *et al*.^14^. For BiFC, DELLA constructs were generated using the same entry clones for LR recombination in the pGPTVII.Bar-C/NYFP vectors containing, respectively, a p35S:CYFP or a p35S:NYFP cassette (http://www.psb.ugent.be/gateway/index.php). For the p35S:NYFP-NF-YA1 cloning, the NF-YA1 entry clone generated in Laloum *et al*.^15^ was recombined into the pGPTVII.Bar-NYFP vector. For p35S:NYFP-NSP1 and p35S:NYFP-NSP2, clones generated in Hirsch *et al*.^16^ were used. For epidermal specific expression, the pFRN-RNAi vector^18^ was cut with EcoRI and SmaI to remove the RNA interference (RNAi) cassette and religated with a linker containing EcoRI and KpnI sites. The p*SlEXT1* promoter was excised from a pGEM-T vector^45^ and cloned into these sites to generate the pFRN-p*SlEXT1* vector. A *della1*-Δ18 PCR fragment amplified from the *pMtDELLA1*:*della1*-Δ18 plasmid with Asc1- and Stu1-containing primers (Supplementary Table 5) was produced and inserted into the p*FRN*-p*SlEXT* vector to generate the p*SlEXT*:*della1*-Δ18 construct. For transactivation assay, the *ARR6* promoter was removed from the pARR6-luciferase plasmid (NCBI accession number EF090414) using BamHI and NcoI restriction enzymes, and replaced by the *ERN1* promoter region defined in Cerri *et al*.^14^, amplified from genomic DNA using BamHI- and NcoI-containing primers (Supplementary Table 5). NSP1, NSP2, NF-YA1 and MtDELLA1 cDNAs were PCR-amplified and cloned in BamHI and StuI restriction sites downstream of a 35S-CaMV promoter in the pHBT plasmid (ref. 67; NCBI accession number EF090408).

### *Agrobacterium rhizogenes* root transformation

Constructs of interest were introduced into the *A. rhizogenes* ARqua1 strain^68^ used for *M. truncatula* root transformation, and transgenic roots were obtained after kanamycin (25 mg l^−1^ for wild-type plants, and 50 mg l^−1^ for the p*ENOD11:GUS* line) selection for 2 weeks^68^. For nodulation experiments, composite plants were transferred onto growth papers (Mega International) on a Fahraeus medium without nitrogen^62^ for 1 week, and transgenic roots were inoculated as described above with a *S. meliloti* or a *S*. *meliloti* strain carrying a *Pro*_Δ*ala*_*:LACZ* transcriptional fusion^63^.

### Histochemical staining

Roots from *pMtDELLA:GUS* composite plants or p*ENOD11:GUS* stable plants were collected at 0 or 1 day post inoculation (dpi) with *S. meliloti* or nodules 21 dpi for histochemical GUS analysis. Histochemical staining for GUS activity was performed for 3 h at 37 °C (ref. 69). After staining, roots or nodules were included in 3% agarose and sliced into 40 μm sections using a VT 1200S vibratome (Leica Microsystems, http://www.leica-microsystems.com/). When necessary, samples or sections were briefly cleared with sodium hypochlorite^69^. Roots and nodules infected by the *S. meliloti* strain expressing the *Pro*_Δ*ala*_:*LACZ* fusion were used for a β-galactosidase staining^62^. Stained samples were observed in bright-field light microscopy (Olympus BX53), and pictures were taken with an Olympus DP73 camera.

### Bimolecular fluorescence complementation

For BiFC assays, the p35S:C/NYFP-MtDELLA1, p35S:C/NYFP-MtDELLA2 and p35S:C/NYFP-MtDELLA3 translational fusions were introduced into an *Agrobacterium tumefaciens* AGL-0 strain, and the p35S:nYFP-NSP1 (ref. 16), p35S:CYFP-NSP2 (ref. 16) and p35S:NYFP-NF-YA1 translational fusions into an *A. tumefaciens* GV3103 strain, and used for transient co-transformation in *N. benthamiana* leaves^70^.

### Co-IP and ChIP

For Co-IP assays, the p35S:*MtDELLA1*-GFP, p35S: *MtDELLA2*-GFP and p35S: *MtDELLA3*-GFP translational fusions were introduced into an *A. tumefaciens* AGL-0 strain and the p35S:HA-*NSP1* (ref. 16), p35S:HA-*NSP2* (ref. 16) and p35S:HA-*NF-YA1* (ref. 15) translational fusions into an *A. tumefaciens* GV3103 strain. After co-transformation in *N. benthamiana* leaves, Co-IPs were performed using Invitrogen Protein A Dynabeads coupled with a α-GFP antibody (Roche 11814460001)^71^. Proteins were separated by SDS–polyacrylamide gel electrophoresis and detected by immunoblotting using a α-GFP antibody (AbCam Ab3277; 1/2,000) or a α-HA antibody (Sigma H6908; 1/5,000) and a α-rabbit horseradish peroxidase secondary antibody (GE Healthcare NA934; 1/10,000). Full blots are shown in Supplementary Fig. 8. For ChIP assays, *M. truncatula* roots transformed with the pMtDELLA1:GFP:*della1-*Δ18 (ref. 32) construct (or GFP as a negative control) were analysed one dpi with *S. meliloti*. IP were performed using a α-GFP antibody (AbCam Ab6556; 1/200), and PCR with primers shown in the Supplementary Table 5.

### Transient activation assays in *Arabidopsis* protoplasts

A 3-day-old *A. thaliana* cell suspension was incubated in a JPL-A medium with 1% (w/v) cellulase RS (Yakult, Tokyo, Japan) and 0.2% (w/v) macerozyme R10 (Yakult)^72^. Protoplasts were collected by centrifugation and washed in the JPL medium supplemented with 0.28 M sucrose. Protoplasts (2.5 10^5^) were transfected with 14 μg of a DNA mixture consisting of 4 μg of the *pERN1:LUC* construct, 9 μg of a combination of the p35S: MtDELLA1, NSP1, NSP2 and/or NF-YA1 plasmids, and 1 μg of the pUBQ10:GUS vector^67^ used as an internal control to normalize the transfection efficiency. Protoplast PEG-transfections (25% (w/v) polyethylene glycol (PEG) 6,000, 450 mM mannitol, 100 mM Ca(NO_3_)_2_ were followed by two washes with a Ca(NO_3_)_2_ 250 mM—JPL-A medium^72^ and incubated overnight at room temperature. Protoplasts were then harvested by centrifugation (100*g*, 5 min), lysed in a protoplasts lysis buffer^67^ and protein extracts were clarified by centrifugation (2,000*g*, 5 min). For GUS assays, protein extracts were incubated 30 min at 37 °C in a MUG substrate mix^72^ and fluorescence was measured with a TECAN Infinite200 fluorimeter. For luciferase assays, protein extracts were mixed with a luciferase assay buffer (20 mM tricine pH 7.8, 5 mM MgCl_2_, 0.1 mM EDTA, 3.3 mM DTT, 270 μM CoA, 500 μM luciferin and 500 μM ATP) and luminescence was immediately measured using a TECAN Infinite200 luminometer.

### Statistical analyses

Statistical analyses were performed with non-parametric tests, Mann–Whitney when *n*=2 independent samples, and Kruskal and Wallis when *n*>2 independent samples.

### Data availability

The authors declare that all data supporting the findings of this study are available within the article and its Supplementary Information Files or are available from the corresponding author upon request.

## Additional information

**How to cite this article:** Fonouni-Farde, C. *et al*. DELLA-mediated gibberellin signalling regulates Nod factor signalling and rhizobial infection. *Nat. Commun.* 7:12636 doi: 10.1038/ncomms12636 (2016).

## Supplementary Material

Supplementary Information Supplementary Figures 1-8, Supplementary Tables 1-5
