# Supplementary material for: DELLA-mediated gibberellin signalling regulates Nod factor signalling and rhizobial infection
Source: Nat Commun. 2016 Sep 2;7:12636. doi: 10.1038/ncomms12636 (PMC5025792; doi:10.1038/ncomms12636)
Supplement: Supplementary Information — Supplementary Figures 1-8, Supplementary Tables 1-5 [file ncomms12636-s1.pdf]

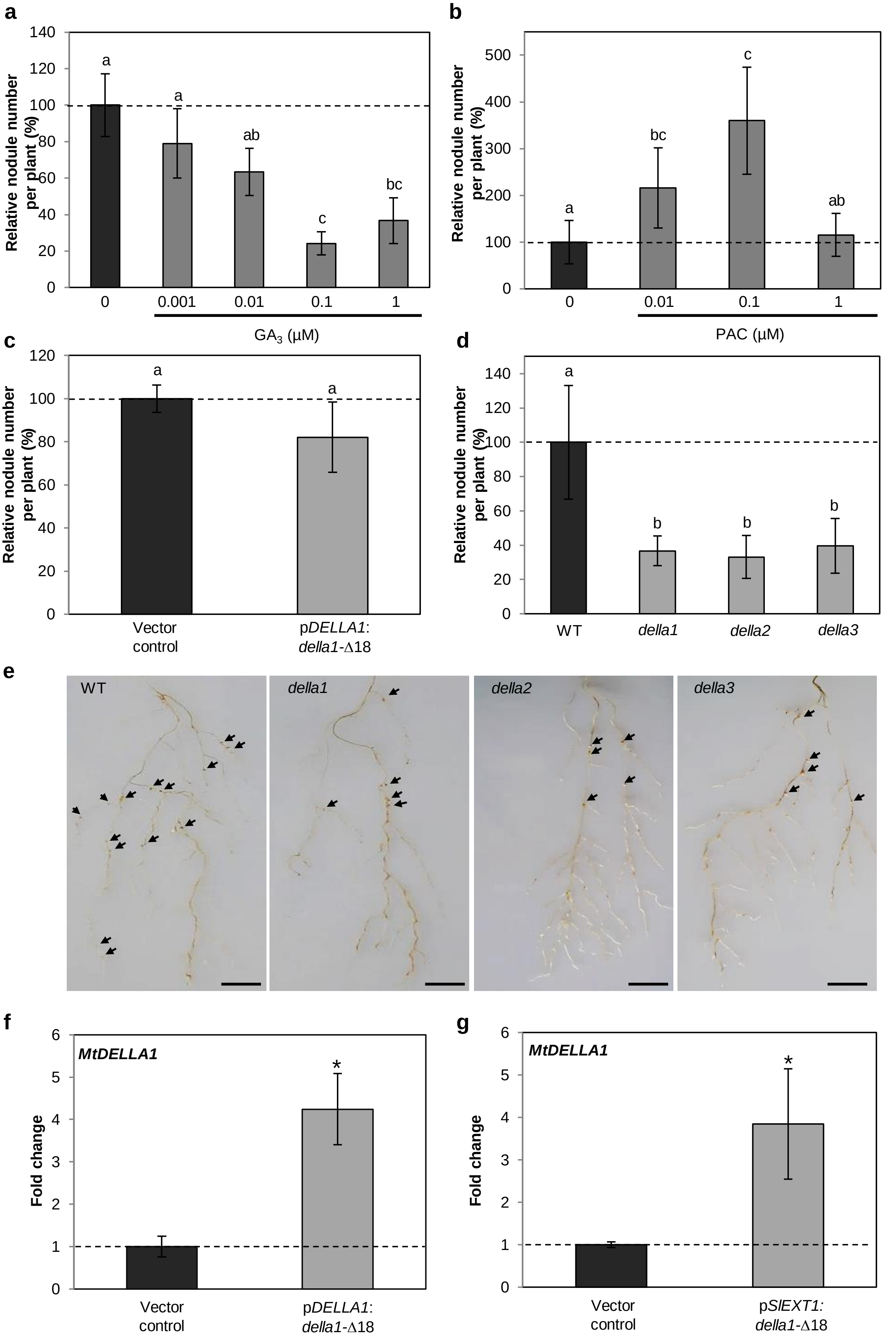

Supplementary Figure 1

Supplementary Figure 1. Gibberellins regulate *Medicago truncatula* nodulation depending on DELLA proteins. (a-b) Relative nodule number per plants in *M. truncatula* plants treated with the gibberellin GA_3 (a) or the GA-biosynthesis inhibitor paclobutrazol (PAC, b) at different concentrations. Results are shown as percentages relatively to the untreated control. (c) Relative nodule number per plants in roots expressing a dominant-active DELLA protein (p*MtDELLA1*:*della1*-Δ18) or the vector control. (d) Relative nodule number in wild-type (WT) and *della* mutants. (e) Representative picture of nodule distribution on wild-type (WT) and *della* mutants root systems. Black arrow-heads indicate symbiotic nodules. Bars=1cm. (f-g). Expression of *MtDELLA1* in root expressing p*MtDELLA1*:*della1*-Δ18 (f) or p*SlEXT:della1*-Δ*18* (g). All transcript levels were normalized relatively to the vector control (the dotted lines indicate a ratio of 1).
In all cases, quantifications were performed 21 days post-inoculation with *Sinorhizobium meliloti* (strain 1021). Error bars represent confidence intervals (α=0.05) for (a-d), and standard deviation for (f) and (g). Letters and asterisks indicate significant differences based on a Kruskal and Wallis test (α<0.05, n=10 plants per condition) for (a), (b) and (d), and a Mann-Whitney test (α<0.05, n>5 plants) for (c), (f) and (g). Results are shown as percentages relatively to the untreated condition in (a), (b), to the WT (d) or to the empty vector control in (c), (f) and (g). The dotted lines indicate a ratio of 100%. One representative example out of two biological replicates is shown.

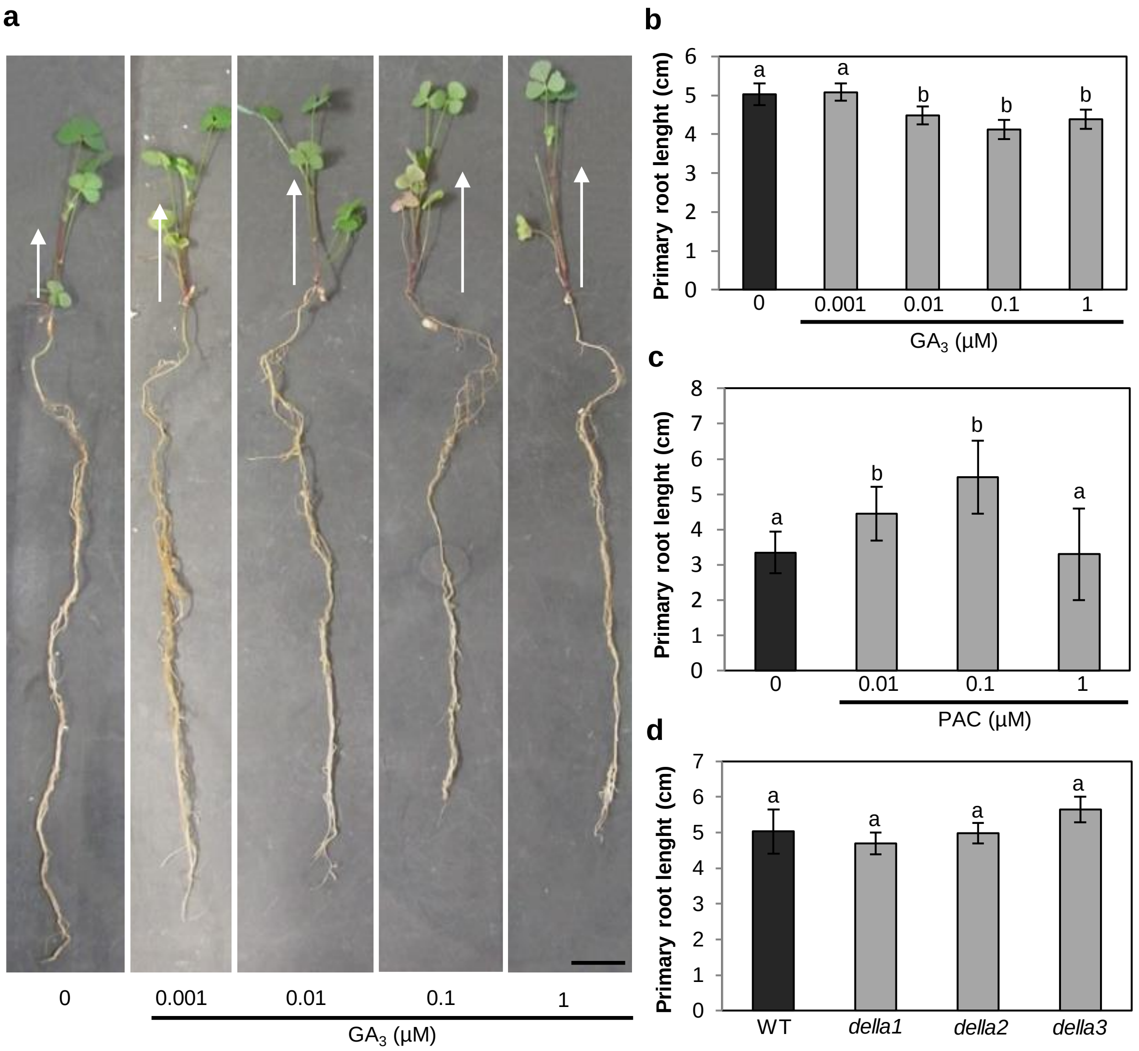

Supplementary Figure 2. Gibberellins regulate *Medicago truncatula* root growth depending on DELLA proteins. (a) Representative phenotypes of *M. truncatula* plants treated with different concentrations of gibberellin (GA_3). A white arrow indicates the length of the two first internodes. Bars=1cm. (b-c) Primary root length of *M. truncatula* plants treated with the gibberellin GA_3 (b) or the GA-biosynthesis inhibitor paclobutrazol (PAC, c) at different concentrations. (d) Primary root length of the three *della* single mutants and of the Wild-Type (WT) control.

Letters indicate significant differences based on a Kruskal and Wallis test ($\alpha<0.05$, n=10 plants per condition). Results are shown as percentages relatively to the untreated condition in (b), (c), or to the WT (d). One representative example out of two biological replicates is shown.

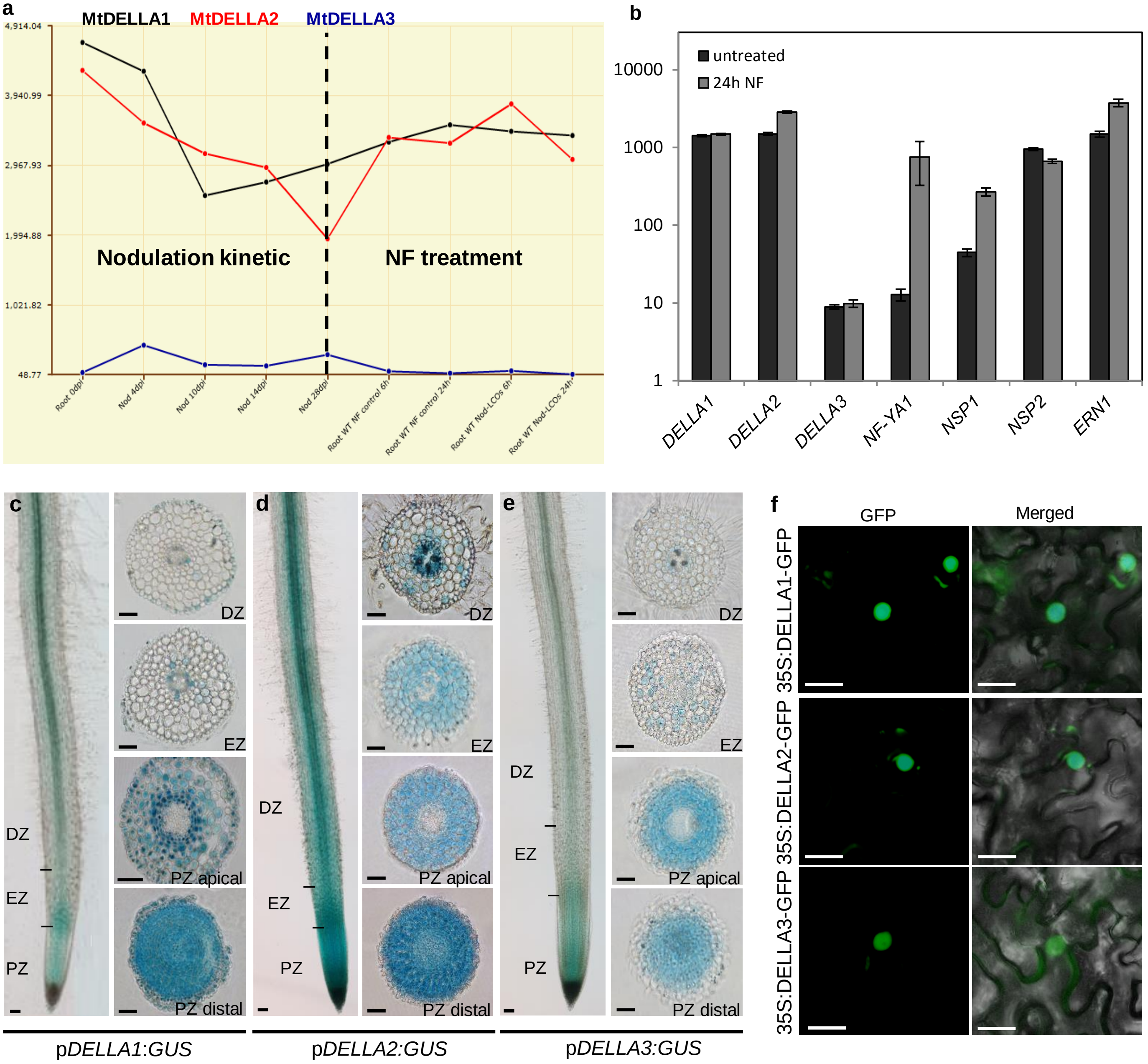

Supplementary Figure 3. Expression patterns of the three *Medicago truncatula MtDELLA* transcripts and MtDELLA proteins. (a) Gene-expression of *MtDELLA1* (black line), *MtDELLA2* (red line) and *MtDELLA3* (blue line) from the *M. truncatula* Gene Expression Atlas (MtGEA database, http://mtgea.noble.org/v2/ [39]). (b) Gene-expression of *MtDELLA1*, *MtDELLA2*, *MtDELLA3, NF-YA1, NSP1, NSP2* and *ERN1* from the 24h NF-treated root hair “infectome” microarray dataset [40]. (c-e) Histochemical analysis of GUS activity of *DELLA* transcriptional fusions in uninoculated roots. Whole roots or 40 µm thick transversal sections of *pMtDELLA1:GUS* (c), *pMtDELLA2:GUS* (d) and *pMtDELLA3:GUS* (e) roots are shown. Root zones are described as follows: PZ, Proliferation Zone; EZ, Elongation Zone; DZ, Differentiation Zone. Bars=100µm. One representative example out of n=20 independent roots is shown. (f) Nuclear localization of the three DELLA-GFP translational fusions expressed from the 35S-CaMV promoter in *Nicotiana benthamiana* leaves. Bars=50µm.

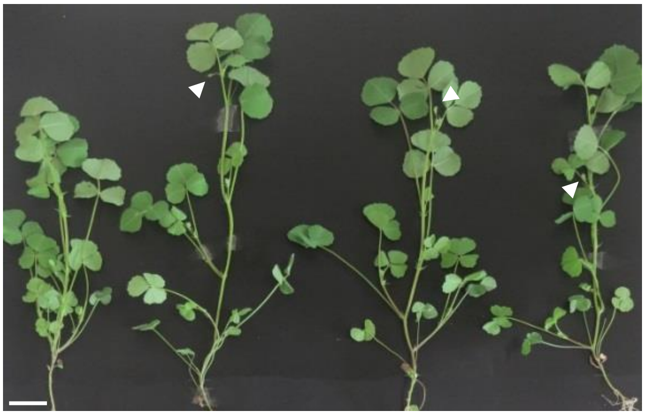

Supplementary Figure 4. Shoot phenotype of the three *Medicago truncatula della* single mutants. Representative picture of Wild-Type (WT) and *della* single mutant shoots. White arrowheads indicate early flowering in *della* mutants. Bars=1cm.

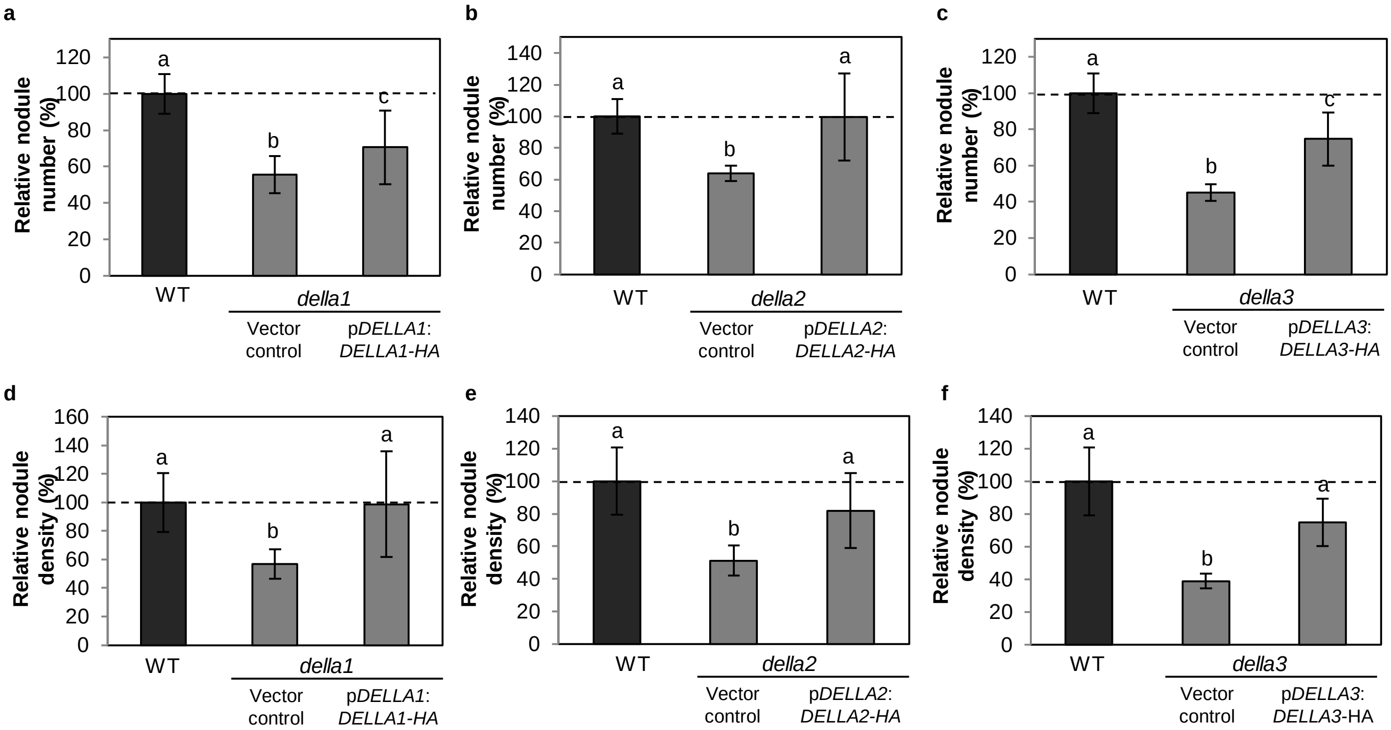

Supplementary Figure 5. Nodulation phenotype of the three *Medicago truncatula della* single mutants complemented with respective p*MtDELLA*-DELLA-HA translational fusions. (a-c) Relative nodule number per plant in Wild-Type (WT) or *della1, della2, della3* single mutants expressing respectively p*MtDELLA1*:DELLA1-HA (a), p*MtDELLA2*:DELLA2-HA (b), p*MtDELLA3*:DELLA3-HA (c) or the empty vector control. (d-f) Relative nodule density (number of nodules per mg of root dry weight) in wild-type (WT) or *della1, della2, della3* single mutants expressing respectively p*MtDELLA1*:DELLA1-HA (d), p*MtDELLA2*:DELLA2-HA (e), p*MtDELLA3*:DELLA3-HA (f) or the empty vector control.
Letters indicate significant differences based on a Kruskal and Wallis test ($\alpha<0.05$, n=8 plants per condition). Results are shown as percentage relatively to the WT empty vector control. One representative example out of two biological replicates is shown. Dotted lines indicate a ratio of 100%.

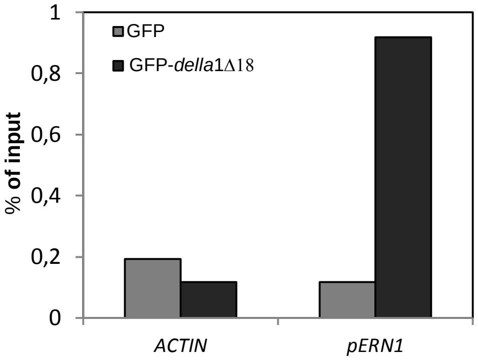

Supplementary Figure 6. MtDELLA1 can associate with the ERN1 promoter. ChIP-qPCR analysis of *della1*-Δ18 binding to the *ERN1* promoter. *M. truncatula* roots transformed with GFP alone or with a *pMtDELLA1*:GFP-*della1*-Δ18 construct were used for ChIP experiments performed with anti-GFP antibodies and primers to amplify by qPCR the *ERN1* promoter region or the *ACTIN11* open reading frame as a negative control. IP values were normalized against those obtained with the Input genomic DNA. One representative example out of two biological replicates is shown.

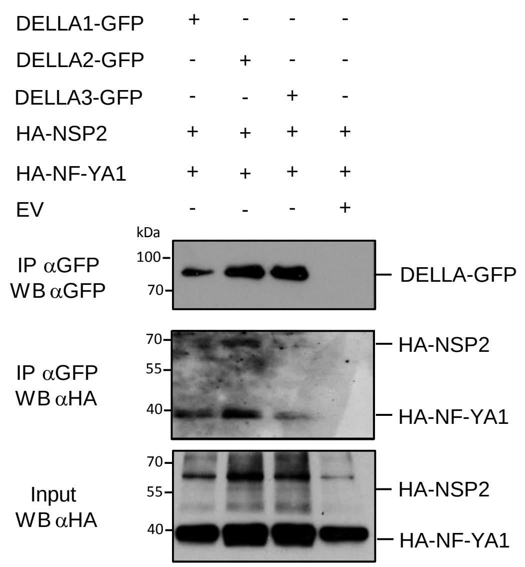

Supplementary Figure 7. NSP2 and NF-YA1 transcription factors can be simultaneously detected following an IP with DELLA proteins. Co-immunoprecipitation of MtDELLA1-GFP, MtDELLA2-GFP or MtDELLA3-GFP proteins expressed in *Nicotiana benthamiana* leaves simultaneously with both HA-NSP2 and HA-NF-YA1 proteins. Blots were revealed with an anti-GFP (upper panel) or an anti-HA (middle and lower panels) antibody. Inputs were used as loading controls.

a

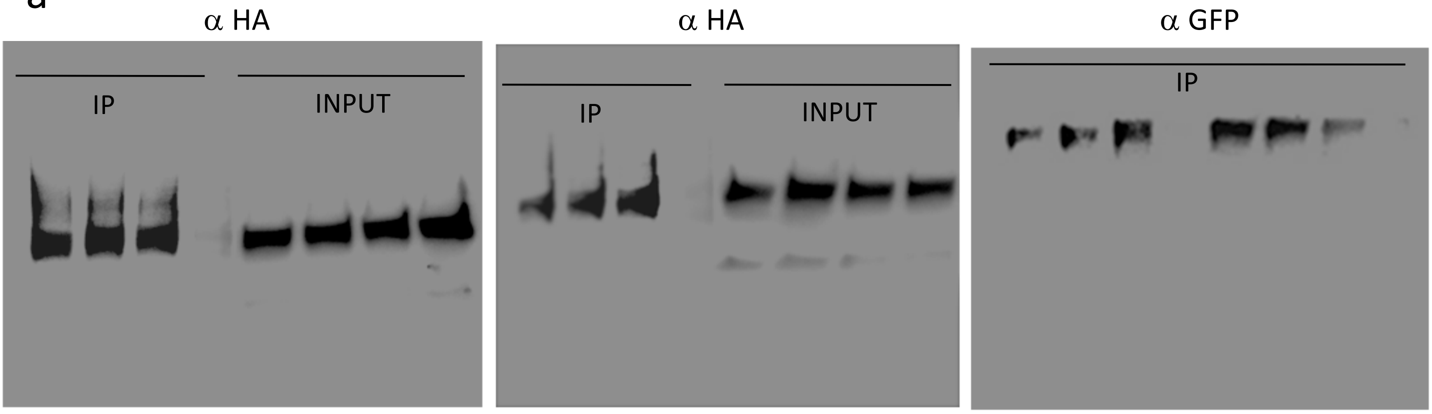

b

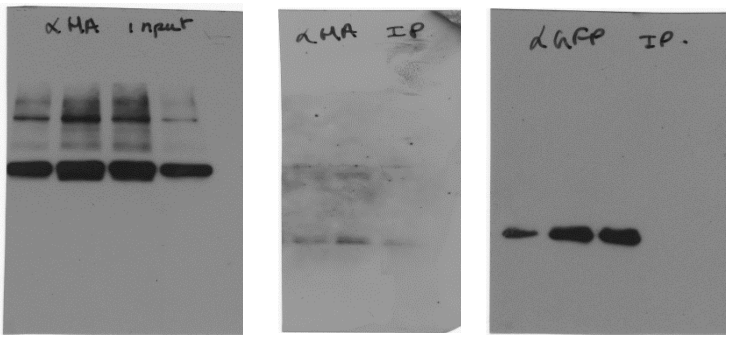

Supplementary Figure 8. Raw data of western blots for co-ImmunoPrecipitation experiments. Blots from Figure 6 and Supplementary Figure 7 are shown respectively in (a) and (b).

GA_3/PAC concentration	Plant number	Root dry weight (mg)	Shoot dry weight (mg)	Number of nodules (per plant)	Nodule density (per mg of root dry weight)	Nodule density (per mg of shoot dry weight)
0	15	15.42 ± 2.04 a	24.58 ± 3.39 a	21 ± 3.61 a	1.41 ± 0.23 a	0.86 ± 0.12 a
0.001µM GA_3	15	15.70 ± 2.46 a	22.50 ± 2.50 a	16.6 ± 3.98 a	1.10 ± 0.29 ab	0.78 ± 0.17 a
0.01 µM GA_3	20	12.50 ± 1.37 ab	17.56 ± 2.82 ab	13.31 ± 2.7 ab	1.04 ± 0.15 ab	0.74 ± 0.14 b
0.1 µM GA_3	15	10.82 ± 1.97 b	15.91 ± 2.90 c	5.09 ± 1.33 c	0.48 ± 0.11 c	0.33 ± 0.08 b
1 µM GA_3	15	11.00 ± 0.99 b	16.91 ± 3.47 bc	7.73 ± 2.63 bc	0.68 ± 0.19 bc	0.48 ± 0.16 b
0	15	14.13 ± 2.26 a	18.93 ± 2.69 ab	10 ± 4.64 a	0.73 ± 0.16 a	0.54 ± 0.12 a
0.01µM PAC	15	14.57 ± 1.69 a	18.07 ± 1.93 a	21.6 ± 8.54 bc	1.43 ± 0.38 bc	1.23 ± 0.27 b
0.1 µM PAC	15	21.62 ± 3.85 b	24.38 ± 3.58 b	36 ± 11.44 c	1.75 ± 0.28 c	1.52 ± 0.23 c
1 µM PAC	15	14.57 ± 1.58 a	17.57 ± 1.75 a	11.5 ± 4.58 ab	0.78 ± 0.12 ab	0.64 ± 0.09 b

Supplementary Table 1. Developmental phenotypes of *Medicago truncatula* plants treated with GA_3 or paclobutrazol at different concentrations. Quantification of root and shoot dry weight (mg), number of nodules (per root) and nodule density (per mg of root or shoot dry weight) of *M. truncatula* plants treated with GA_3 (0.001µM to 1µM) or paclobutrazol (PAC, 0.01µM to 1µM) compared to untreated controls (+/- Confidence Intervals, α=0.05). Letters indicate significant differences based on a Kruskal and Wallis test ($\alpha<0.05$).

Affymetrix probe	Gene name	MEAN FI%	MEAN FIID%	MEAN FIIP%	MEAN IZ%	MEAN ZIII%
Mtr.37355.1.S1_at	*MtDELLA1*	23.8	29.7	13.3	17.5	15.7
Mtr.10469.1.S1_at	*MtDELLA2*	24.3	23.8	19.0	17.6	15.4
Mtr.11244.1.S1_at	*MtDELLA3*	40.8	18.5	16.4	14.8	9.5

Supplementary Table 2. Expression of the three *MtDELLA* genes in different nodule zones. Expression of the three *MtDELLA* genes in the different nodule zones based on the laser dissection analysis performed in Roux *et al.*[41] (SYMBIMICS database, http://iant.toulouse.inra.fr/symbimics/). FI=Meristematic zone, FIID=Distal differentiation/infection zone, FIIP= Proximal differentiation/infection zone, IZ=Interzone, ZIII= Nitrogen fixation zone.

Genotype	Plant number	Root dry w eight (mg)	Shoot dry w eight (mg)	Number of nodules (per plant)	Nodule density (per mg of root dry w eight)	Nodule density (per mg of shoot dry w eight)
Wild Type	20	19.79 ± 1.72 a	24.55 ± 2.43 a	15.58 ± 4.26 a	0.81 ± 0.10 a	0.68 ± 0.27 a
della 1	15	15.73 ± 2.49 a	23.09 ± 3.71 a	4.83 ± 1.13 b	0.34 ± 0.09 b	0.22 ± 0.09 b
della 2	15	20.60 ± 4.28 a	17.4 ± 5.96 a	6.80 ± 2.59 b	0.33 ± 0.11 b	0.25 ± 0.11 b
della 3	15	15.20 ± 5.09 a	24.4 ± 0.48 a	5.20 ± 2.09 b	0.35 ± 0.10 b	0.21± 0.09 b

Supplementary Table 3. Developmental phenotypes of *della1*, *della2* and *della3* single mutants. Quantifications of root and shoot dry weight (mg), number of nodules (per root) and nodule density (per mg of root or shoot dry weight) in *M. truncatula della1*, *della2* and *della3* single mutants or Wild- Type (WT) plants (+/- Confidence Intervals, $\alpha=0.05$). Letters indicate significant differences based on a Kruskal and Wallis test ($\alpha<0.05$).

Genotype	Plant number	Number of infection events per plant
Wild Type (7 d.p.i)	10	18.70 ± 2.25 a
della 1 (7 d.p.i)	10	4.20 ± 0.70 b
della 2 (7 d.p.i)	10	3.80 ± 0.76 b
della 3 (7 d.p.i)	10	4.50 ± 0.73 b
GA treatment (7 d.p.i)	15	2.76 ± 0.74 b
Vector control (7 d.p.i)	10	46.90 ± 3.38 a
p*DELLA1:della1*- Δ18 (7 d.p.i)	10	103.17 ± 12.40 b
Vector control (3 d.p.i)	15	2.87 ± 0.69 a
p*SlEXT1:della1*- Δ18 (3 d.p.i)	15	4.6 ± 1.14 b
Vector control (7 d.p.i)	15	41.10 ± 10.04 a
p*SlEXT1:della1*- Δ18 (7 d.p.i)	15	71.67 ± 21.67 b

Supplementary Table 4. Infection phenotypes in response to a GA_3 treatment or genetic modifications of the gibberellin signaling pathway. Quantifications of infection events in *M. truncatula della1*, *della2* and *della3* single mutants, or in Wild-Type (WT) plants treated with GA_3, or in roots expressing *della1*-Δ18 from the p*MtDELLA1* promoter (p*MtDELLA1:della1*-Δ18) or from the p*SlEXT1* epidermal promoter (p*SlEXT1:della1*-Δ18). As indicated in the first column, quantifications were performed at different days post inoculation (dpi) with a *Sinorhizobium meliloti* strain expressing a *LACZ* reporter (+/- Confidence Intervals, α=0.05). Letters indicate significant differences based on a Kruskal and Wallis test (α<0.05) for rows 1 to 5 (corresponding to a single experiment) or a Mann-Whitney test (α<0.05) for rows 6 to 11 (corresponding to other independent experiments).

**Mutant genotyping**				
				**Pair of primers used for**
LTR4	5’-TACCGTATCTCGGTGCTACA-3’	*Wt-DELLA1-Rev*	5’-TAGCTTCGTTACAAACCGGTGC-3’	**Tnt1 insertion amplification**
LTR4	5’-TACCGTATCTCGGTGCTACA-3’	*Wt-DELLA2-Rev*	5’-AACTCCGGTCACCGGAAAC-3	
LTR6	5’-GCTACCAACCAAACCAAGTCAA-3’	*Wt-DELLA3-Rev*	5’-ACGTTGCACGGAATCTGCACAGGTC-3’	
Wt-DELLA1-Fw	5’-GTTGCAAAGCATCAGTATTGTCC-3’	*Wt-DELLA1-Rev*	5’-TAGCTTCGTTACAAACCGGTGC-3’	**Endogenous WT locus amplification**
Wt-DELLA2-Fw	5’-TCAGTGCGAAACCACCACTGAG-3’	*Wt-DELLA2-Rev*	5’-AACTCCGGTCACCGGAAAC-3	
Wt-DELLA3-Fw	5’-GTTATTGCGTGGAAGAGAATC-3’	*Wt-DELLA3-Rev*	5’-ACGTTGCACGGAATCTGCACAGGTC-3’	
**qRT-PCR**				
ACTIN-Fw	5’-ACGAGCGTTTCAGATG-3’	*ACTIN-Rev*	5’-ACCTCCGATCCAGACA-3	**qPCR analysis**
RBP1-Fw	5’-AGGGGCAAGTTCCTTCATTT-3’	*RBP1-Rev*	5’-GGTAGAAGTGCTGGCTCAGG-3’	
ERN1-Fw	5’-GGAAGATGGTGCTGTTGCTT-3'	*ERN1-Rev*	5’-TGTTGGATTGTGAACCTGACTC-3'	
ENOD11-Fw	5’-ATCCCACAATATGCCTCCAA-3’	*ENOD11-Rev*	5’-AGGAAGTGGTGGCTTTAGCA-3’	
GA20Ox-Fw	5’-GTGTCCATTAAGTGATAAAGTGG-3’	*GA20Ox-Rev*	5’-GCTTTTACATTGAACCCACTTGGC-3’	
GA2Ox-Fw	5’-CTGCAAATCTGTCTCACTGATGG-3’	*GA2Ox-Rev*	5’-GTTGTGTCAGCCAAAACCCTATGC-3’	
NSP1-Fw	5'-GCGATTTCGCCACTGGATTC	*NSP1-Rev*	5'-CAGCCTCGCCTTCCATCATT-3'	
NSP2-Fw	5’-TTCATTGGAGGCTGGTTTTC-3’	*NSP2-Rev*	5’-CACCCACCTCTTCAACCCTA-3’	
NIN-Fw	5’-GCAATGTGGGGATTTAGAGATT-3’	*NIN-Rev*	5’-GGAAGATTGAGAGGGGAAGCTT-3’	
NFYA1-Fw	5’-TCGGATCTACTGTCCACTCTTTGG-3’	*NFYA1-Rev*	5’-TTGGCATGACGATACCGTGTC-3’	
Chip-pERN1-Fw	5'-TCGGATCTACTGTCCACTCTTTGG-3'	*Chip-pERN1-Rev*	5'-TTGGCATGACGATACCGTGTC-3'	
***In Situ* Hybridization**				
DELLA1-AS-Fw	5’-GCTAAGATCATACTCAGAATC-3’	*DELLA1-AS-T7-Rev*	5’-TGTAATACGACTCACTATAGGGCGCTAAGATCATACTCAGAATC-3’	**Anti Sense probes (AS)**
DELLA2-AS-Fw	5’-GGTATGGATGAACTCTTAGC-3’	*DELLA3-AS-T7-Rev*	5’-TGTAATACGACTCACTATAGGGCCGTTTCCTGGAAGTAAACTCG-3’	
DELLA3-AS-Fw	5’-GTGCGATCGTGAACTCATCCAGC-3’	*DELLA3-AS-T7-Rev*	5’-TGTAATACGACTCACTATAGGGCGCATGTGGACAAGTTTTATTGC-3’	
CA1-AS-Fw	5'-TGATCGACTTGCTAAAGGCC-3'	*CA1-AS-T7-Rev*	5'-TGTAATACGACTCACTATAGGGCCCAAAGCTTGAATTCTCCAC-3'	
CA1-S-T7-Fw	5'-TGTAATACGACTCACTATAGGGCCAACAAGAACCCGGAACTGT-3'	*CA1-S-Rev*	5'-GAGCAGAACCATCCTCTGGA-3'	**Sense probe (S)**
**DELLA-GUS transcriptional fusion**				
DELLA1-GUS-Fw	5’-CACCGAGTATGCCAAAACACGATGAC-3'	*DELLA1-GUS-Rev*	5’-GTTGATTAAATTGGTGAAGTG-3’	***MtDELLA* promoter amplification**
DELLA2-GUS-Fw	5’-CACCGAGTATGCAGTCATAAAGTAC-3’	*DELLA2-GUS-Rev*	5’-CTTTCACTTTTTCAGTTTTCTC-3’	
DELLA3-GUS-Fw	5’-CACCCGTAATGCCTGAATCTCTACG-3’	*DELLA3-GUS-Rev*	5’-AGAATTCAAAACTTAAAACACAG-3’	
**DELLA-GFP translational fusion**				
cDNA-DELLA1-Fw	5’-CACCATGAAGAGAGAACACCAAG-3’	*cDNA-DELLA1-Rev*	5’-GGATCACTTGGACTCATTTTGTG-3’	***MtDELLA* cDNA amplification**
cDNA-DELLA2-Fw	5’-CACCATGAAGAGAGAGCATAAGC-3’	*cDNA-DELLA2-Rev*	5’-GGATCAGTGCGAAACCACCACTG-3’	
cDNA-DELLA3-Fw	5’-CACCATGGAAATAGTTTCAGATTC-3’	*cDNA-DELLA3-Rev*	5’-GGATCAACAATCAAAACGCAGTG-3’	
***della1*- Δ18 epidermal specific expression**				
DELLA1-Asc1-Fw	5’-TA**GGCGCGCC**ATGAAGAGAGAACACCAAGAAAG-3’	*DELLA1-Stu1-Rev*	5’-TC**AGGCCT**ACACTTGGACTCATTTTGTGGAAGCTTC-3’	***della1*- Δ18 amplification**
**transactivation assay**				
DELLA1_BamHI_Fw	5'-TGAC**GGATCC**ATGAAGAGAGAACACCAAGAAAGTTT-3'	*DELLA1_StuI_Rev*	5'-GATC**AGGCCT**CTTGGACTCATTTTGTGGAAGC-3'	**cDNA amplification**
NSP1_BamHI_Fw	5'-TGAC**GGATCC**ATGACTATGGAACCAAATCCAAC-3'	*NSP1_SmaI_Rev*	5'-GATC**CCCGGG**TCTGGTTGTTTATCCAGTTTCC-3'	
NSP2_BamHI_Fw	5'-TGAC**GGATCC**ATGGATTTGATGGACATGGAT-3'	*NSP2_StuI_Rev*	5'-GATCG**AGGCCT**TAAATCAGAATCTGAAGAAGAACAAGT-3'	
NF-YA1_BamHI_F	5'-TGAC**GGATCC**ATGGCTATGCAACCTGTTTATCTT-3'	*NF-YA1_StuI_Rev*	5'-GATC**AGGCCT**TGGTTCCAGCAGCTGTCTG-3'	
pERN1_BamHI_Fw	5'-TGCA**GGATCC**GAATTCAACTTAATGACTCTCGAGTC-3'	*pERN1_NcoI_rev*	5'-GATC**CCATGG**CAAAGTTTTTTCAAGAAATTGTATTTG-3'	

Supplementary Table 5. List of primers. Each horizontal row corresponds to pairs of Forward (Fw) and Reverse (Rev) primers. LTR4 and LTR6 are *TnT1* retrotransposon-specific primers used to genotype *della* mutants. T7 indicates the sequence of the T7 promoter used to synthesize RNA probes for *in situ* hybridisation. S= sense, AS=AntiSense. Underlined sequences correspond to restriction sites added for cloning (see Methods).
